# The missing base molecules in atmospheric acid–base nucleation

**DOI:** 10.1093/nsr/nwac137

**Published:** 2022-07-25

**Authors:** Runlong Cai, Rujing Yin, Chao Yan, Dongsen Yang, Chenjuan Deng, Lubna Dada, Juha Kangasluoma, Jenni Kontkanen, Roope Halonen, Yan Ma, Xiuhui Zhang, Pauli Paasonen, Tuukka Petäjä, Veli-Matti Kerminen, Yongchun Liu, Federico Bianchi, Jun Zheng, Lin Wang, Jiming Hao, James N Smith, Neil M Donahue, Markku Kulmala, Douglas R Worsnop, Jingkun Jiang

**Affiliations:** State Key Joint Laboratory of Environment Simulation and Pollution Control, School of Environment, Tsinghua University, Beijing 100084, China; Institute for Atmospheric and Earth System Research / Physics, Faculty of Science, University of Helsinki, Helsinki 00014, Finland; State Key Joint Laboratory of Environment Simulation and Pollution Control, School of Environment, Tsinghua University, Beijing 100084, China; Institute for Atmospheric and Earth System Research / Physics, Faculty of Science, University of Helsinki, Helsinki 00014, Finland; Aerosol and Haze Laboratory, Beijing Advanced Innovation Center for Soft Matter Science and Engineering, Beijing University of Chemical Technology, Beijing 100029, China; Collaborative Innovation Center of Atmospheric Environment and Equipment Technology, Nanjing University of Information Science and Technology, Nanjing 210044, China; State Key Joint Laboratory of Environment Simulation and Pollution Control, School of Environment, Tsinghua University, Beijing 100084, China; Institute for Atmospheric and Earth System Research / Physics, Faculty of Science, University of Helsinki, Helsinki 00014, Finland; Laboratory of Atmospheric Chemistry, Paul Scherrer Institute, Villigen 5232, Switzerland; Institute for Atmospheric and Earth System Research / Physics, Faculty of Science, University of Helsinki, Helsinki 00014, Finland; Institute for Atmospheric and Earth System Research / Physics, Faculty of Science, University of Helsinki, Helsinki 00014, Finland; Center for Joint Quantum Studies and Department of Physics, School of Science, Tianjin University, Tianjin 300350, China; Collaborative Innovation Center of Atmospheric Environment and Equipment Technology, Nanjing University of Information Science and Technology, Nanjing 210044, China; Key Laboratory of Cluster Science, Ministry of Education of China, School of Chemistry and Chemical Engineering, Beijing Institute of Technology, Beijing 100081, China; Institute for Atmospheric and Earth System Research / Physics, Faculty of Science, University of Helsinki, Helsinki 00014, Finland; Institute for Atmospheric and Earth System Research / Physics, Faculty of Science, University of Helsinki, Helsinki 00014, Finland; Institute for Atmospheric and Earth System Research / Physics, Faculty of Science, University of Helsinki, Helsinki 00014, Finland; Aerosol and Haze Laboratory, Beijing Advanced Innovation Center for Soft Matter Science and Engineering, Beijing University of Chemical Technology, Beijing 100029, China; Institute for Atmospheric and Earth System Research / Physics, Faculty of Science, University of Helsinki, Helsinki 00014, Finland; Collaborative Innovation Center of Atmospheric Environment and Equipment Technology, Nanjing University of Information Science and Technology, Nanjing 210044, China; Shanghai Key Laboratory of Atmospheric Particle Pollution and Prevention (LAP^3^), Department of Environmental Science and Engineering, Fudan University, Shanghai 200433, China; State Key Joint Laboratory of Environment Simulation and Pollution Control, School of Environment, Tsinghua University, Beijing 100084, China; Chemistry Department, University of California, Irvine, CA 92697, USA; Center for Atmospheric Particle Studies, Carnegie Mellon University, Pittsburgh, PA 15213, USA; Department of Chemistry, Carnegie Mellon University, Pittsburgh, PA 15213, USA; Institute for Atmospheric and Earth System Research / Physics, Faculty of Science, University of Helsinki, Helsinki 00014, Finland; Institute for Atmospheric and Earth System Research / Physics, Faculty of Science, University of Helsinki, Helsinki 00014, Finland; Aerodyne Research Inc., Billerica, MA 01821, USA; State Key Joint Laboratory of Environment Simulation and Pollution Control, School of Environment, Tsinghua University, Beijing 100084, China

**Keywords:** new particle formation, acid-base nucleation, aerosol, polluted urban environment

## Abstract

Transformation of low-volatility gaseous precursors to new particles affects aerosol number concentration, cloud formation and hence the climate. The clustering of acid and base molecules is a major mechanism driving fast nucleation and initial growth of new particles in the atmosphere. However, the acid–base cluster composition, measured using state-of-the-art mass spectrometers, cannot explain the measured high formation rate of new particles. Here we present strong evidence for the existence of base molecules such as amines in the smallest atmospheric sulfuric acid clusters prior to their detection by mass spectrometers. We demonstrate that forming (H_2_SO_4_)_1_(amine)_1_ is the rate-limiting step in atmospheric H_2_SO_4_-amine nucleation and the uptake of (H_2_SO_4_)_1_(amine)_1_ is a major pathway for the initial growth of H_2_SO_4_ clusters. The proposed mechanism is very consistent with measured new particle formation in urban Beijing, in which dimethylamine is the key base for H_2_SO_4_ nucleation while other bases such as ammonia may contribute to the growth of larger clusters. Our findings further underline the fact that strong amines, even at low concentrations and when undetected in the smallest clusters, can be crucial to particle formation in the planetary boundary layer.

## Teaser

Forming an (H_2_SO_4_)_1_(amine)_1_ cluster is the rate-limiting step in atmospheric H_2_SO_4_-amine nucleation, which governs the fast formation of new particles from gaseous precursors.

## INTRODUCTION

New particle formation (NPF) events occur frequently in various atmospheric environments [1,2]. These newly formed particles, after subsequent growth, constitute a major source of cloud condensation nuclei [3,4]. To assess the influences of NPF on radiative forcing [5], it is of fundamental importance to understand the NPF mechanisms. The first and key step of NPF is nucleation, during which gaseous precursors form the smallest stable clusters that are more likely to grow into large particles than to evaporate [6,7]. Among the reported nucleation mechanisms for atmospheric environments [8–17], acid–base nucleation is unique for its effectiveness in forming neutral clusters at ambient temperatures and typical precursor concentrations in the planetary boundary layer. Laboratory experiments [8,18–20] and theoretical studies [21,22] have shown that many bases can stabilize H_2_SO_4_ clusters and drive fast NPF. The atmosphere is a complex system containing various bases such as amines and ammonia. Identifying key base molecules in the small H_2_SO_4_ clusters from a large pool of candidate base vapors [23] is pivotal to understanding atmospheric H_2_SO_4_-base nucleation.

Measurements of cluster composition via chemical ionization mass spectrometry can provide evidence for bases involved in NPF [8,20,24,25]; however, some base molecules are missing from the observed cluster signals obtained from these instruments. For instance, theoretical calculations based on quantum chemistry indicate that the acid–base ratio of most H_2_SO_4_-base clusters is 1:1 [21,26,27], as also hypothesized in some laboratory studies [18,20,24,28]. In contrast, signals from ambient H_2_SO_4_ clusters contain fewer base molecules with many containing no base molecules at all [10,29]. This is likely a measurement artifact.

Due to the missing base molecules, the detected cluster signals cannot be used to exclusively identify the key bases. To be measured in a mass spectrometer, clusters must either be charged via chemical ionization or be naturally charged in the atmosphere. In either case, a cluster, especially a key cluster formed in the rate-limiting steps of H_2_SO_4_ nucleation, may lose base molecules upon charging. This is because acid and base molecules nucleate effectively by forming strong hydrogen bonds (as well as other intermolecular forces). For instance, charging a neutral (H_2_SO_4_)_1_(base)_1_ cluster by deprotonation converts the acid–base pair to an unstable base–base pair, with HSO_4_^−^ as a very strong base. The unstable (HSO_4_^−^)_1_(base)_1_ cluster subsequently loses the base molecule and hence is detected as a bare HSO_4_^−^ ion. Charging by clustering with a reagent ion also affects cluster stability and may cause base evaporation [27]. Additionally, charged clusters may also lose base molecules inside the mass spectrometer due to collisions between cluster and carrier gas molecules [30].

For much the same reason, matching the composition of large atmospheric H_2_SO_4_ clusters to those measured in laboratory experiments cannot exclusively identify the key base. With many candidate bases in the atmosphere, the bases measured in large clusters may not be the key bases for forming the smallest clusters.

Comparing H_2_SO_4_ concentrations and particle formation rates [9,10,19,31] also cannot exclusively identify the key base. The same particle formation rate may be driven by either weak bases with high concentrations or strong bases with concentrations even lower than the instrumental detection limit [22].

Understanding nucleation mechanisms at the molecular level is also substantially challenged by the missing bases from detected atmospheric H_2_SO_4_ cluster signals. As previously mentioned, nucleation pathways and the most relevant stable clusters can be predicted with cluster kinetics and quantum calculations [7,32]. It has been suggested that H_2_SO_4_ and the key base form (H_2_SO_4_)_1_(base)_1_ during nucleation [21,26,27]. Considering the uncertainties of quantum chemistry results, theoretical predictions require experimental verification; this verification of cluster composition and rate-limiting steps has been largely precluded by the missing bases in those measurements. Well-controlled laboratory experiments [18,20] have demonstrated that a small cluster containing two H_2_SO_4_ and one or two strong bases can already be stable against evaporation. However, the stability of various (H_2_SO_4_)_1_(base)_1_ clusters remains uncertain. Because (H_2_SO_4_)_1_(base)_1_ clusters have not been detected in the atmosphere, a previous study proposed that stabilization of (H_2_SO_4_)_2_ by adding a strong base to it may be the rate-limiting step for atmospheric nucleation [9].

To summarize, the missing bases in the smallest H_2_SO_4_ clusters are key to a better understanding of atmospheric H_2_SO_4_-base nucleation. In this study, we provide strong evidence for the existence and importance of the missing bases in atmospheric H_2_SO_4_ clusters using data from atmospheric measurements and laboratory experiments as well as process model simulations. We demonstrate that the first and rate-limiting step of neutral H_2_SO_4_-base nucleation, which is often referred to as the step in which the ‘critical cluster’ is formed in classical nucleation theory [33], is to form (H_2_SO_4_)_1_(amine)_1_, instead of the formation and subsequent stabilization of (H_2_SO_4_)_2_. In polluted urban environments such as Beijing and Shanghai, a considerable fraction (up to 70%) of the H_2_SO_4_ molecules is clustered with amines, with dimethylamine (DMA) as the key base. Depending on vapor concentrations and temperature, formation of (H_2_SO_4_)_1_(DMA)_1_ is either a major rate-limiting step or nucleation occurs close to the H_2_SO_4_ + DMA amine-saturation limit without a free energy barrier. Right after nucleation, the dominant cluster growth mechanism depends on the available DMA and other bases. At a high DMA concentration, cluster growth is mainly driven by the addition of (H_2_SO_4_)_1_(DMA)_1_. At a low DMA concentration, synergy with other weaker but more abundant bases, e.g. ammonia or other amines, may enhance H_2_SO_4_ cluster growth and hence increase the particle formation rate.

## RESULTS

To identify the key base(s) for nucleation in polluted atmospheres, we first focus on H_2_SO_4_-DMA nucleation and demonstrate that the key step is the formation of the undetected (H_2_SO_4_)_1_(DMA)_1_. After that, we discuss H_2_SO_4_-base nucleation in the complex atmosphere with various candidate bases including DMA, ammonia and other amines. Following convention [8,9,34], we refer to the measured H_2_SO_4_ clusters as n-mers according to the number of constituent H_2_SO_4_ molecules. For instance, all clusters containing two H_2_SO_4_ molecules and any number of bases are referred to as H_2_SO_4_ dimers. The acid–base cluster composition is written as A_m_B_n_, where A represents H_2_SO_4_, B represents a certain base and the subscripts indicate the numbers of molecules contained in a given cluster.

### Measured cluster composition

**Figure 1.**
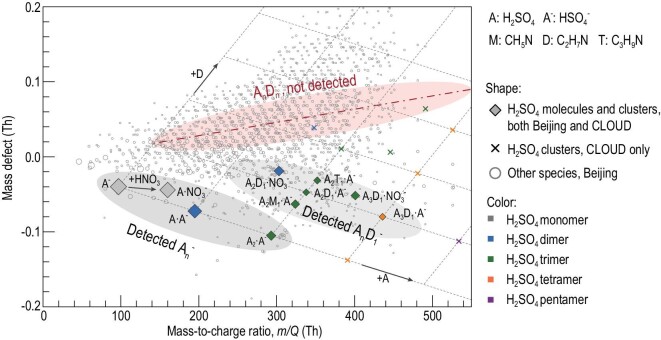
Molecules and clusters measured during atmospheric acid–base NPF events from H_2_SO_4_ and amines. Neutral molecules and clusters were negatively charged by NO_3_^−^ and HN_2_O_6_^−^ during the detection using chemical ionization mass spectrometry. The detected H_2_SO_4_ clusters in urban Beijing contained 0–1 amine molecules. The detected amine molecules in H_2_SO_4_ clusters were mainly dimethylamine (DMA, D) and trimethylamine (T). Deprotonated or NO_3_^−^ clustered A_n_D_n_ clusters were not detected in urban Beijing. Clusters composed of more than five H_2_SO_4_ or two DMA molecules have been reported for laboratory experiments [20,24,35] with high DMA concentrations, such as the CLOUD chamber experiments. All the H_2_SO_4_-DMA clusters detected in urban Beijing were also detected in these laboratory experiments. The colors of markers for H_2_SO_4_ clusters indicate the number of H_2_SO_4_ molecules contained in each cluster. The sizes of H_2_SO_4_ clusters and other species measured in urban Beijing indicate their signal intensities, yet they follow a different size scale in order to emphasize the H_2_SO_4_ clusters. The dashed grid indicates the numbers of H_2_SO_4_ and DMA molecules contained in each deprotonated H_2_SO_4_-DMA cluster. The dash-dotted line indicates the deprotonated A_n_D_n_ clusters, which were not detected. The shaded ellipses are drawn to guide the eye.

We observed neutral H_2_SO_4_ clusters in urban Beijing during NPF events using advanced online chemical ionization mass spectrometers. As shown in Fig. 1, the signals of atmospheric H_2_SO_4_ monomers and dimers contain no base molecules, while trimers and tetramers contain up to one amine molecule. The detected dominant species of monomers, dimers and trimers are A_1_^−^, A_2_^−^ and A_3_^−^, respectively, and tetramers were mainly detected as A_4_D_1_^−^ (D for DMA). In urban Beijing, we have not identified larger neutral cluster signals containing more than four H_2_SO_4_ molecules or more than one amine molecule. The naturally charged H_2_SO_4_ clusters, detected using an atmospheric pressure interface time-of-flight mass spectrometer, show much the same pattern, with no base molecules present in H_2_SO_4_ monomers and dimers (Fig. S1 in Supplementary Data online). The H_2_SO_4_ clusters measured in urban Beijing have also been observed in laboratory experiments [20,24,35]. Due to the more abundant H_2_SO_4_ and DMA vapors in these laboratory experiments, large clusters containing more H_2_SO_4_ and DMA molecules were detected. Despite these, the H_2_SO_4_ monomers therein were also observed only in the form of bare H_2_SO_4_ molecules, A_1_, i.e. the base molecule was missing.

The directly measured H_2_SO_4_ monomer signals could be misinterpreted as an indication that most H_2_SO_4_ monomers are bare molecules (or perhaps hydrated clusters with undetected water). This would suggest that H_2_SO_4_-DMA nucleation is initialized by the clustering between two bare H_2_SO_4_ molecules and the addition of a DMA to an A_2_ [9], or that A_3_ is the rate-limiting step in stabilizing clusters against evaporation. However, such interpretations based on the directly observed signals from H_2_SO_4_ clusters are very likely flawed. As discussed in the introduction, base molecules are missing from clusters measured under atmospheric conditions. This is mainly because a neutral cluster has to be charged during detection. The additional charge or reagent ion converts a stable H_2_SO_4_-DMA cluster into a potentially unstable cluster. As the base strength of HSO_4_^−^ is higher than DMA, the unstable cluster tends to become stable by evaporating DMA molecules [27]. The collision between a cluster and carrier gases in the mass spectrometer may also cause DMA evaporation [30]. In particular, the ubiquitous, large fractions of bare A_1_^−^, A_2_^−^ and A_3_^−^ are artifacts of the detection process. The evidence is given below.

### Evidence for the existence of (H_2_SO_4_)_1_(amine)_1_

In this section, we show experimental evidence that a large fraction of A_1_D_1_ clusters contributes to the signal of ambient H_2_SO_4_ monomers using the measured H_2_SO_4_ dimer concentration and its variation with DMA concentration and temperature. The measured dimer concentration and its variation can only be well explained by the existence of a large fraction of A_1_D_1_ clusters in ambient H_2_SO_4_ monomers, with A_1 _+ D_1_ → A_1_D_1_ being the rate-limiting step for nucleation.

In order to show the influence of DMA concentration and temperature, we eliminate the strong dependence of dimer concentration on monomer concentration by comparing dimer concentration to its amine-saturation limit. The amine-saturation limit is herein referred to as the dimer concentration calculated with the assumption that each collision between two H_2_SO_4_ monomers generates a stable H_2_SO_4_ dimer, i.e. base concentration is not a rate-limiting factor. Hence, dimer concentration at the amine-saturation limit is approximately the theoretical maximum for a given monomer concentration and cluster scavenging rate.

H_2_SO_4_ dimers are mainly formed via

$$\begin{equation*}
{A}_1 + {A}_1 \to {A}_2,
\end{equation*}$$

$$\begin{equation*}
{A}_1 + {A}_1{D}_1 \to {A}_2{D}_1,
\end{equation*}$$

and

$$\begin{equation*}
{A}_1{D}_1 + {A}_1{D}_1 \to {\rm{ }}{A}_2{D}_2.
\end{equation*}$$

The latter two reactions should be efficient as A_2_D_1_ and A_2_D_2_ are stable against evaporation [20,22,36]. However, an A_2_ cluster needs to be further stabilized by adding one base molecule [9]; hence, the effectiveness of dimer formation via A_1_ + A_1_ is governed by the stability of A_2_ against evaporation and its association rate with stabilizing bases.

**Figure 2.**
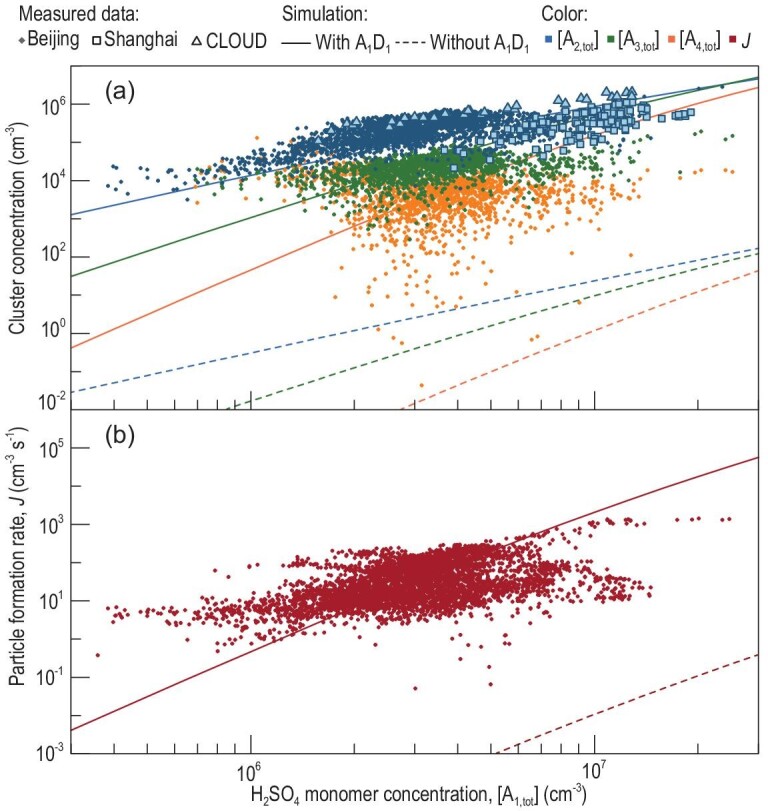
(a) H_2_SO_4_ cluster concentrations and (b) particle formation rates as a function of H_2_SO_4_ monomer concentration. The measured H_2_SO_4_ cluster concentrations and NPF rate were consistent with the simulation results for which forming an (H_2_SO_4_)_1_(amine)_1_ cluster is the first and critical step of NPF. The amine molecule is mainly dimethylamine (D) for the moderate stability of A_1_D_1_ against evaporation. The simulation without forming A_1_D_1_ yields H_2_SO_4_ cluster concentrations and a particle formation rate that are orders of magnitude lower than those measured in urban Beijing. The measured data in urban Beijing were collected during daytime (9:00–16:00 local time) NPF events with a 5-min temporal resolution. The H_2_SO_4_ dimer concentrations for Shanghai [10] and CLOUD experiments [28] were previously reported. The concentrations of clusters containing the same number of H_2_SO_4_ molecules were summed up, e.g. [A_2,tot_] is the total concentration of measured or simulated H_2_SO_4_ dimers containing any number of base molecules. The influencing factors, such as the cluster loss rate characterized by the condensation sink (CS), amine concentrations and temperature (*T*), affect cluster concentrations and formation rate, and their slope versus [A_1,tot_] [54, 68]. These influences are not corrected in this figure because they are minor compared to the differences between the simulation results with and without forming A_1_D_1_. The curves were simulated for [DMA] = 1.8 ppt (∼4.7 × 10^7^ cm^−3^), CS = 0.011 s^−1^ and *T* = 281 K, which are the medians of the measured data.

The measured high concentrations of atmospheric H_2_SO_4_ clusters provide indirect but strong experimental evidence for the existence of A_1_D_1_ rather than only A_1_. As shown in Fig. 2, if all the H_2_SO_4_ monomers existed in the form of A_1_ but not A_1_D_1_, H_2_SO_4_ dimer, trimer and tetramer concentrations would be orders of magnitude lower than the measured values. This shows that the mechanism with dimer stabilization via A_2_ + D_1_ [9] as the rate-limiting step is not effective for nucleation due to the instability of A_2_ [22]. This inefficiency is also supported by chamber experiments with H_2_SO_4_ and ammonia [8], which have shown that despite the stability of ammonia-stabilized A_2_ [22] and a high ammonia concentration, the dimer stabilization mechanism is insufficient to explain the measured cluster concentrations (see Fig. S2). In contrast, if A_1_D_1_ constituted a large fraction of H_2_SO_4_ monomers and nucleation is rate-limited by forming A_1_D_1_, this could explain the cluster concentrations in Beijing [37,38], Shanghai [10] and also in Cosmics Leaving OUtdoor Droplets (CLOUD) chamber experiments [28].

**Figure 3.**
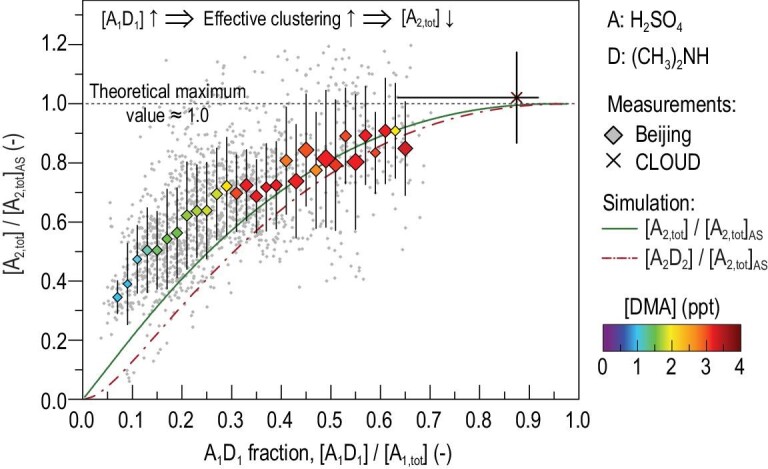
H_2_SO_4_ dimer concentration as a function of theoretical (H_2_SO_4_)_1_(amine)_1_ concentration. The measured H_2_SO_4_ dimer concentration ([A_2,tot_]) is consistent with the simulated A_1_D_1_ concentration, where D represents DMA. With a given H_2_SO_4_ monomer concentration ([A_1,tot_]), increasing [A_1_D_1_] improves the effectiveness of clustering between H_2_SO_4_ monomers in terms of forming stable H_2_SO_4_ dimers. [A_2,tot_] approaches its maximum, characterized by the amine-saturation limit ([A_2,tot_]_AS_), as the simulated ratio of [A_1_D_1_] to [A_1,tot_] increases. The simulation also shows that [A_2_D_2_] comprises a major fraction in [A_2,tot_]. Hence, [A_2,tot_] can be used as an agent for the missing A_1_D_1_ (see Methods). The Beijing data are shown in edged markers that represent the mean value of the measured 5-min resolution data group by [A_1_D_1_]/[A_1,tot_], with [A_1_D_1_] calculated using Equation (2). The marker size indicates the mean CS of each group. The variation bars indicate the standard deviation for each group. The data from CLOUD chamber studies were reported in ref. [20]. The [A_1_D_1_]/[A_1,tot_] shown by the crossed marker and horizontal variation bar was estimated for [DMA] = 20 ppt and 5–32 ppt, respectively. In this figure, [A_1,tot_] and the [A_2,tot_] shown in markers are measured data. [A_1_D_1_] and the [A_2,tot_] shown in curves are simulation results. [A_2,tot_]_AS_ is calculated using the measured [A_1,tot_] and coagulation sink.

The measured dependence of H_2_SO_4_ dimer concentration on DMA concentration provides further strong support for the existence of A_1_D_1_ as well as its importance. As shown in Fig. 3, the measured H_2_SO_4_ dimer concentration increases with an increasing DMA concentration, which can be well explained by an increasing A_1_D_1_ fraction in the H_2_SO_4_ monomers. That is, with the same H_2_SO_4_ monomer concentration, there are more A_1_D_1_ clusters at a higher DMA concentration, driving more efficient H_2_SO_4_ dimer formation towards its amine-saturation limit.

**Figure 4.**
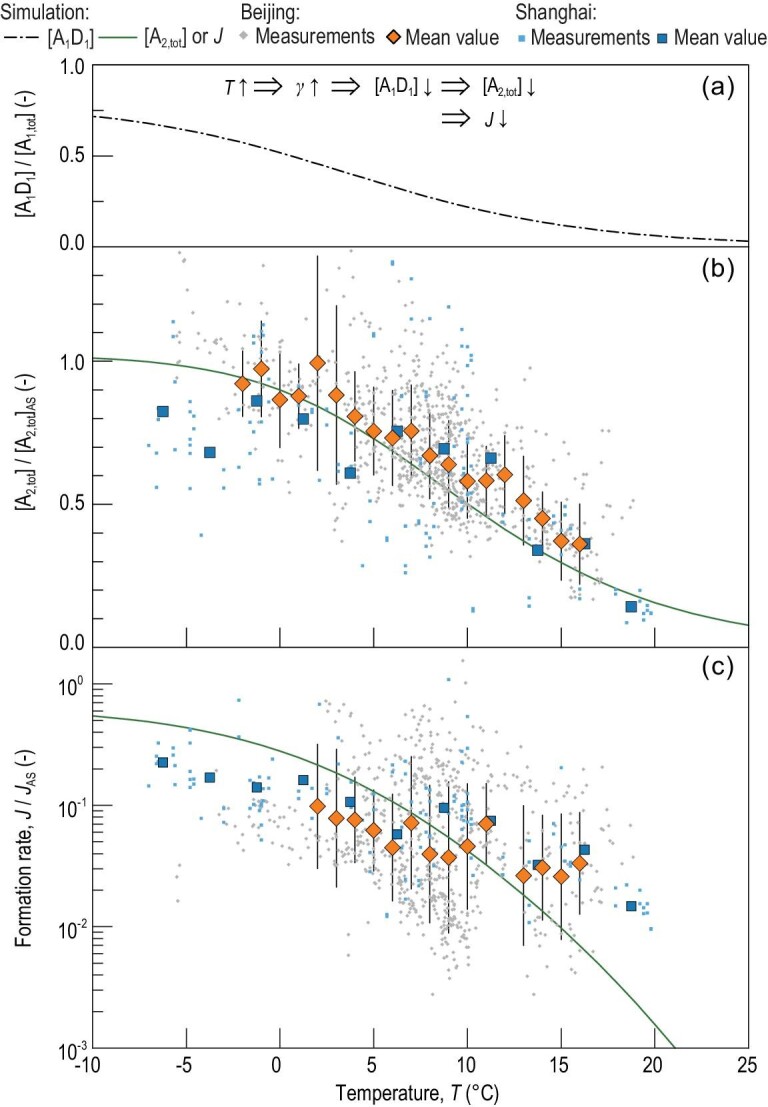
H_2_SO_4_ dimer concentration and particle formation rate as a function of temperature. (a) The simulated fraction of A_1_D_1_ in H_2_SO_4_ monomers, where D is dimethylamine; (b) normalized H_2_SO_4_ dimer concentration ([A_2,tot_]) as a function of temperature; (c) normalized formation rate (*J*) of 1.4 nm particles as a function of temperature. The evaporation rate of A_1_D_1_ (*γ*) increases with an increasing temperature, which decreases A_1_D_1_ concentration. As a result, [A_2,tot_] and *J* decrease with an increasing temperature. The temperature dependence of [A_1_D_1_] is consistent with the measured data in urban Beijing and Shanghai. [A_2,tot_]_AS_ and *J*_AS_ are the total H_2_SO_4_ dimer concentration and particle formation rate, respectively, at the amine-saturation limit (see Fig. 3 and Methods). The curves were simulated at the median CS (0.011 s^−1^) and the median amine concentration for the Beijing data set. The big markers are the median values of measured raw data grouped by temperature. The variation bars indicate the lower and upper quartiles for each group. The formation rate was calculated for 1.4 nm particles. The measured [A_2,tot_] for urban Shanghai, shown in (b), was scaled with a multiplicative factor of 3 (see Methods) but this scaling does not affect the measured temperature dependence of [A_2,tot_].

The measured temperature dependence of the H_2_SO_4_ dimer concentration gives further evidence for the existence of A_1_D_1_ and its significant fraction in ambient H_2_SO_4_ monomers. Figure 4b shows that H_2_SO_4_ dimer concentrations decrease with increasing temperature [39] for the measured NPF events in Beijing and Shanghai [10] (see also Fig. S3). This temperature dependence is consistent with the existence of A_1_D_1_. The evaporation rate of A_1_D_1_ increases with an increasing temperature, and the A_1_D_1_ fraction in monomers thus decreases significantly (Fig. 4a), causing the decreasing H_2_SO_4_ dimer concentration (Fig. 4b).

The DMA-dependent A_1_D_1_ fraction in H_2_SO_4_ monomers is also very consistent with the CLOUD experiments [20]. During those experiments, high DMA concentrations (>5 ppt) and a low temperature (278 K) forced a high A_1_D_1_ fraction that drove dimer formation close to its amine-saturation limit, and hence nucleation was found to be insensitive to DMA concentration in the (DMA-saturated) experimental conditions.

The experimental evidence above confirms the existence of A_1_D_1_ and its importance in H_2_SO_4_-DMA nucleation. For the observed NPF events in urban Beijing, the median DMA concentration was 1.8 ppt and the A_1_D_1_ fraction H_2_SO_4_ in monomers could be as high as 70% with 4-ppt DMA.

The existence of ambient A_1_D_1_ clusters indicates moderate evaporation of A_1_D_1_, which is consistent with the evaporation rate obtained from quantum chemistry and laboratory experiments. We use cluster kinetics to derive the temperature-dependent evaporation rates of A_1_D_1_ from atmospheric measurements (see Methods). These rates are consistent with reported quantum chemical results [22,36] (Fig. S4). Laboratory experiments have also estimated upper limits for the evaporation rate of A_1_D_1_ clusters using high DMA concentrations (>5 ppt at 278 K) [28,35], yet our atmospheric measurements provide consistent but lower evaporation rates at lower atmospheric DMA concentrations.

The measured NPF rate provides support for the above experimental evidence. The rapid formation of stable H_2_SO_4_ dimers from A_1_D_1_ enables a rapid formation of new particles. As shown in Fig. 2, the simulated NPF rate with a large A_1_D_1_ fraction in H_2_SO_4_ monomers is consistent with the measured formation rate in Beijing; otherwise, the simulated formation rate would be orders of magnitude lower. In Fig. 4c, NPF rates in Beijing and Shanghai decrease with increasing temperature, which can be explained by the decreasing stability of A_1_D_1_ against evaporation as a function of increasing temperature. Besides, the existence of A_1_D_1_ is also consistent with the positive correlation between NPF rate and DMA concentration for NPF in urban Beijing [37].

### Atmospheric nucleation with various bases

The above results also demonstrate that forming A_1_D_1_ is the key rate-limiting step for atmospheric H_2_SO_4_-DMA nucleation. The key role of A_1_D_1_ for H_2_SO_4_-DMA nucleation can be generalized to H_2_SO_4_ nucleation with other bases: forming A_1_B_1_ is often the rate-limiting step. H_2_SO_4_ can nucleate close to its amine-saturation limit only when a considerable fraction of H_2_SO_4_ monomers exist in the form of A_1_B_1_ clusters. We use this criterion below to show that DMA is the missing key base from various candidates for nucleation in urban Beijing.

In addition to C_2_-amine (probably DMA), we detected gas-phase methylamine (MA), C_3_-amine (probably trimethylamine, TMA), C_4_-amine and ammonia in urban Beijing [29] using mass spectrometry. The measured neutral H_2_SO_4_ clusters also contain TMA (T) in the form of A_4_T_1_ (Fig. 1). C_4_-amine and ammonia were detected in the naturally charged H_2_SO_4_ clusters (Fig. S1). The measured cluster signals are consistent with the measured gas-phase bases. Other candidate bases for H_2_SO_4_-base nucleation, such as ethylene diamine [19], were not detected in the gas-phase or H_2_SO_4_ clusters.

**Figure 5.**
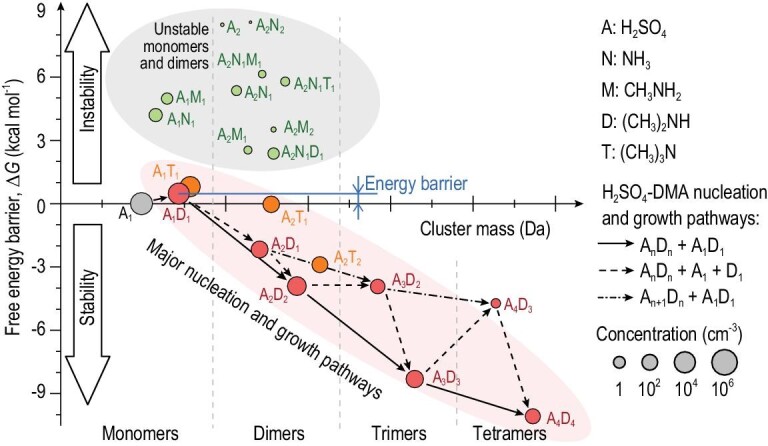
Gibbs free energy barrier of H_2_SO_4_ clusters with various base molecules. Forming stable clusters against evaporation corresponds to a low free energy barrier (Δ*G*). Nucleation and cluster growth mainly follow the pathways with low Δ*G*. A positive Δ*G* corresponds to a free energy barrier that limits the nucleation rate and cluster concentrations. With typical concentrations of gaseous precursors in urban Beijing, forming A_1_D_1_ and A_1_T_1_ is more effective than forming A_2_, A_1_M_1_ and A_1_N_1_, yet the moderate evaporation rates of A_1_D_1_ and A_1_T_1_ clusters (indicated by their Δ*G*) still limits NPF. Because of the considerable A_1_D_1_ concentration and low Δ*G* of A_n_D_n_ clusters (n > 1), the coagulation between A_1_D_1_ and A_n_D_n_ clusters is a major growth pathway. The results for this figure were simulated for the following conditions: [A_1,tot_] = 3.4 × 10^6^ cm^−3^, [N] = 1.1 ppb (∼2.9 × 10^10^ cm^−3^), [M] = 0.2 ppt (∼5.2 × 10^6^ cm^−3^), [D] = 1.8 ppt (∼4.7 × 10^7^ cm^−3^), [T] = 0.5 ppt (∼1.3 × 10^7^ cm^−3^), CS = 0.011 s^−1^ and *T* = 281 K. These values are the medians for the Beijing data set during daytime (9:00–16:00) NPF events. The growth pathway from A to A_2_D_1_ is not shown because it is identical to the pathway from A_1_D_1_ to A_2_D_1_. The shaded ellipses and hollow arrows are drawn to guide the eye.

The free energy barrier of H_2_SO_4_ monomers and dimers containing different bases shows that despite the contributions from other amines, forming A_1_D_1_ is still the key rate-limiting step for fast H_2_SO_4_-base nucleation in this complex urban atmosphere. An increasing value of free energy upon adding one molecule corresponds to a significant evaporation rate, and a decreasing value indicates that the growth of the cluster is faster than its evaporation. As shown in Fig. 5, DMA governs the formation of stable H_2_SO_4_ dimers because the free energy of A_1_D_1_ is the lowest among the H_2_SO_4_ monomers stabilized with other measured bases. For the typical conditions in urban Beijing, an H_2_SO_4_ molecule needs to overcome a low free energy barrier to become an A_1_D_1_ cluster. After that, the A_1_D_1_ cluster grows into a particle via pathways with consecutively descending free energy.

In addition to DMA, TMA may have a secondary contribution to nucleation in urban Beijing. Considering the free energy of A_1_D_1_ and A_1_T_1_ [21] (Fig. 5), TMA is comparable to DMA in forming stabilized H_2_SO_4_ dimers [18]. After accounting for the base concentrations, however, we find that the contribution of TMA does not affect the key role of DMA in nucleation in urban Beijing.

Weak bases, such as MA and ammonia (N), are thermodynamically unfavorable for atmospheric nucleation. Figure 5 shows the high free energy of A_1_M_1_ and A_1_N_1_, despite the high ammonia concentration (∼1 ppb during the observed NPF events in urban Beijing). As a result, the unstable A_1_M_1_ or A_1_N_1_ clusters with low concentrations significantly limit H_2_SO_4_-MA and H_2_SO_4_-ammonia nucleation and they cannot explain the measured high H_2_SO_4_ dimer concentration and particle formation rate.

Strong bases with low concentrations are kinetically unfavorable for atmospheric nucleation despite their ability to form very stable clusters. We did not detect bases stronger than DMA (except HSO_4_^−^), such as diamines [19], in the measured neutral and naturally charged H_2_SO_4_ clusters or the gas phase, indicating their concentrations are below the instrumental detection limit. A low base concentration, especially when it is lower than the H_2_SO_4_ concentration, may kinetically limit the fraction of A_1_B_1_ in H_2_SO_4_ monomers [19] even though A_1_B_1_ might be thermodynamically favorable for nucleation. For instance, taking HSO_4_^−^ as a base, the free energy of A_2_^−^ is extremely low [27,40], yet forming A_2_^−^ in the atmosphere is kinetically limited by the low ambient HSO_4_^−^ concentration (typically <10 ions/cm^3^ for urban Beijing). In other words, ion-induced nucleation even at the ion-pair formation rate of a few particles cm^−3^ s^−1^ is simply not competitive with the high nucleation rates we observe (*J* >> 10 cm^−3^ s^−1^).

The temperature dependence of the H_2_SO_4_ dimer concentration also provides evidence that DMA, rather than some unidentified strong base, is the missing key base for H_2_SO_4_ nucleation in urban Beijing. Although the dimer concentration could reach the same value with a low concentration of an unidentified strong base [19,22], it is unlikely that this strong base would cause the similar temperature dependence of the dimer concentration (see Fig. S5).

## DISCUSSION

We have presented strong evidence that DMA is the missing key base for H_2_SO_4_-base nucleation in polluted urban environments, and the formation of A_1_D_1_ up to a considerable fraction (70% for urban Beijing) in H_2_SO_4_ monomers is the rate-limiting step. The existence of A_1_D_1_ is robust despite the fast formation and depletion of H_2_SO_4_ monomers. We find that ambient A_1_D_1_ reaches its pseudo-steady-state concentration within minutes due to its short overall lifetime, and this pseudo-steady-state is not sensitive to a rapid change in the production rate of A_1_ molecules (see Fig. S6).

Although DMA is found to dominate nucleation in urban Beijing, other bases, particularly strong bases, may also contribute to nucleation at conditions such as low DMA concentrations and high temperatures. As shown in Fig. 3, the formation of A_1_D_1_ cannot fully explain the measured dimer concentration at a low A_1_D_1_ fraction (<0.1) in H_2_SO_4_ monomers. Further, with proper environmental conditions, other strong bases such as TMA and diamines may play important roles if they are more abundant than DMA [18,19].

Despite their minor roles in nucleation, ammonia and abundant weak amines may have synergistic effects on the initial growth of H_2_SO_4_-DMA clusters. Especially at relatively low DMA concentrations, a large fraction of H_2_SO_4_ monomers are bare (or hydrated) H_2_SO_4_ molecules (Figs 3 and 5). This bare H_2_SO_4_ may not effectively contribute to the initial growth of H_2_SO_4_-DMA clusters due to the potential high evaporation rates of A_n+1_D_n_ clusters (n ≥ 2). For instance, some quantum chemical results [22] have suggested that A_4_D_3_ might be more likely to evaporate back into A_3_D_3_ than to grow into A_4_D_4_. That is, H_2_SO_4_ might not contribute effectively to the growth of ambient A_3_D_3_. However, with a high concentration of ammonia, a potentially unstable A_4_D_3_ cluster can be rapidly stabilized by ammonia and form A_4_D_3_N_1_ (N stands for ammonia) [41]. This synergy enhances cluster growth via the condensation of A_1_ molecules and also increases the particle formation rate (see Fig. S7), which is consistent with laboratory results [42].

However, the synergy of weak bases should not boost particle formation when DMA is sufficient. When most H_2_SO_4_ monomers exist in the form of A_1_D_1_ at a high DMA concentration, A_n_D_n_ + A_1_D_1_ is the governing pathway for cluster growth [21,43] (Fig. S8) and the particle formation rate is limited by the other losses (e.g. coagulation loss) rather than cluster evaporation. In this case, adding weak bases such as ammonia should not significantly increase the formation rate of sub-2 nm particles [8]. Note that base substitution [41,44] is still possible via the fast formation and evaporation of unstable clusters such as A_3_D_3_N_1_. Since these unstable clusters are more likely to evaporate than grow, observing a minor fraction of ammonia molecules in large H_2_SO_4_ clusters [20,24] does not necessarily indicate a contribution of ammonia to the growth rate of the H_2_SO_4_-DMA clusters.

In addition to bases, water molecules may contribute to stabilizing H_2_SO_4_ clusters and they are also missing from the detected cluster signals [45]. However, no significant dependence of H_2_SO_4_ dimer concentration and particle formation rate on water vapor concentration was observed after accounting for the influences of other factors. This insignificant effect of hydration on H_2_SO_4_-DMA nucleation is consistent with quantum chemical calculations [21].

Although the nucleation rate of H_2_SO_4_ and base is close to the amine-saturation limit (Fig. 3), sub-2 nm particle growth is mainly driven by the measured H_2_SO_4_ monomers with only a minor contribution from dimers and larger H_2_SO_4_ clusters. This is because the high coagulation sink in polluted urban environments suppresses the cluster concentrations (Fig. 2), which is different from low-sink conditions such as in chamber experiments [46].

To summarize, the key cluster for ambient H_2_SO_4_-base nucleation is missing from the cluster signals detected using state-of-the-art instruments. Using consistent evidence from atmospheric measurements, laboratory experiments and a process model based on cluster kinetics and quantum chemistry, we demonstrate that (H_2_SO_4_)_1_(amine)_1_ must be the missing key cluster for the polluted atmosphere, with DMA as the key base and forming (H_2_SO_4_)_1_(DMA)_1_ as the rate-limiting step. During H_2_SO_4_-amine nucleation, a substantial fraction of H_2_SO_4_ monomers exist in the form of (H_2_SO_4_)_1_(amine)_1_ and the uptake of (H_2_SO_4_)_1_(amine)_1_ is a major mechanism for the initial growth of clusters. Other abundant weak bases may have synergistic effects on the growth of H_2_SO_4_-DMA clusters by stabilizing larger H_2_SO_4_-DMA clusters before their evaporation and thus enhance the particle formation rate.

Further, we argue that atmospheric nucleation should be characterized using the rate-limiting factors and steps, instead of only the concept of the ‘critical cluster’. According to the definition of classical nucleation theory [7], the undetected key (H_2_SO_4_)_1_(DMA)_1_ cluster is the ‘critical cluster’ at typical atmospheric conditions for urban Beijing. It is worth clarifying that the ‘critical cluster’ depends on the vapor concentrations. For H_2_SO_4_-DMA nucleation with a high DMA concentration (20 ppt), as shown in Fig. S8, nucleation occurs without a free energy barrier and the particle formation rate is mainly limited by the coagulation sink. Conversely, in conditions with very low H_2_SO_4_ and DMA concentrations, the ‘critical cluster’ can be H_2_SO_4_ dimers or larger clusters, despite the extremely low evaporation rates of A_2_D_1_ and A_2_D_2_ [20,22,36]. As a result, it is crucial for both laboratory and theoretical studies to better represent atmospheric conditions or properly apply the results to atmospheric conditions.

## MATERIALS AND METHODS

### Atmospheric measurements

The data used in this study were obtained from atmospheric measurements in urban Beijing and urban Shanghai. The NPF process from gaseous precursors to particles was measured at both sites. The Beijing site is located at the west campus of the Beijing University of Chemical Technology (39°56^′^ N, 116°17^′^ E), ∼500 m away from the west 3rd Ring Road. The data set used for this study was from 16 January 2018 to 13 March 2019, with gaseous amines and ammonia measurements available after 20 October 2018. Neutral gaseous H_2_SO_4_ molecules and clusters, C_1_–C_3_ amines, ammonia, and aerosol size distributions were measured. H_2_SO_4_ molecules and clusters were measured using high-resolution chemical ionization time-of-flight mass spectrometers (HToF-CIMS, Aerodyne Research, Inc.) with NO_3_^−^ and HN_2_O_6_^−^ as the reagent ions [47]. Amines and ammonia were measured using a modified HToF-CIMS with H_3_O^+^ or its hydrated clusters as the reagent ions [48]. Since isomers cannot be differentiated by an HToF-CIMS, the C_2_-amines and C_3_-amines were taken as DMA and TMA, respectively. Therefore, the measured DMA and TMA concentrations might be overestimated. However, since ethylamine is less effective than DMA in forming A_1_B_1_ clusters [49], a major fraction of the measured C_2_-amines are likely to be DMA. Other bases such as ethylene diamine were not identified. Aerosol size distributions were measured using a diethylene glycol scanning mobility particle spectrometer (DEG-SMPS) for sub-5 nm aerosols [50–52] and a particle size distribution system for 3 nm–10 μm aerosols [53]. Ambient temperature was monitored using a weather station (AWS310, Vaisala Inc.). Details of the measurement site, instruments and measurement uncertainties have been reported previously [37,38,54,55].

The Shanghai data set was reported in a previous study [10]. The Shanghai site is located at the campus of Fudan University (31°18^′^ N, 121°30^′^ E), ∼100 m from the Middle Ring Road. Neutral gaseous H_2_SO_4_ and aerosol size distributions were measured simultaneously from 4 December 2015 to 10 February 2016. H_2_SO_4_ molecules and clusters were measured using an HToF-CIMS with NO_3_^−^ and HN_2_O_6_^−^ as the reagent ions. Aerosol size distributions were measured using a particle size magnifier [56] (Airmodus Inc.) for 1–3 nm aerosols, and two scanning mobility particle spectrometers (SMPSs) (TSI Inc.) for 3–65 nm aerosols and 14–736 nm aerosols. No measured C_2_-amine concentration was available during this period, yet it was measured at the same site in August 2015 using an HToF-CIMS with protonated ethanol as reagent ions [57]. Ambient temperature was recorded at a site ∼5 km away.

The concentrations of H_2_SO_4_ dimers, trimers and tetramers were evaluated using the same calibration factor for H_2_SO_4_ monomers. The mass-dependent transmission efficiency of HToF-CIMS was calibrated and corrected [58]. Due to the fragmentation of H_2_SO_4_ clusters within HToF-CIMS [30], the H_2_SO_4_ dimer, trimer and tetramer concentrations might be underestimated. For the Beijing data set, the underestimation of H_2_SO_4_ dimer concentration was estimated to be ∼30%, which is close to the estimated values in a laboratory experiment [20]. This value is within the uncertainty range of H_2_SO_4_ measurements and it does not affect the conclusions based on both the absolute value of H_2_SO_4_ dimers and its dependence on amine concentration and temperature. The measured H_2_SO_4_ dimer concentration in Shanghai was multiplied by three, yet only its temperature dependence is used for discussion in this analysis.

The formation rate of 1.4 nm particles (*J*_1.4_) in urban Beijing was retrieved from measured aerosol size distributions using a population balance formula [59] improved for intensive NPF events in polluted atmospheric environments. The *J*_1.4_ in urban Shanghai was calculated from the reported *J*_1.7_ using particle growth rate and the coagulation sink [60].

### Theory

Here we present a theory with reasonable approximations to illustrate the importance of (H_2_SO_4_)_1_(amine)_1_ to the formation of clusters containing two or more H_2_SO_4_ molecules, though the figures in the main text are obtained using numerical simulation without these approximations. A dimensionless parameter, *η*, is defined to characterize the ratio of (H_2_SO_4_)_1_(base)_1_ to H_2_SO_4_ monomers. For a simplified system containing only H_2_SO_4_ (A), a certain species of base (B), and aerosols, the population balance equation for A_1_B_1_ is

(1) \begin{eqnarray*} \frac{{{\rm{d}}\left[ {{{\rm{A}}}_1{{\rm{B}}}_1} \right]}}{{{\rm{d}}t}} &=& {\beta }_{{\rm{AB}}}\left[ {{{\rm{A}}}_1} \right]\left[ {{{\rm{B}}}_1} \right] \nonumber\\ && -\, \left( {\gamma \left( T \right) + {\rm{CS}}} \right)\left[ {{{\rm{A}}}_1{{\rm{B}}}_1} \right], \end{eqnarray*}

where [A_1_B_1_], [A_1_] and [B_1_] are the concentrations (cm^−3^) of A_1_B_1_ clusters, bare A_1_ molecules and B_1_ molecules, respectively; *t* is time (s); *β*_AB_ is the coagulation coefficient (cm^3^ s^−1^) between an A_1_ molecule and a B_1_ molecule; *γ* is the evaporation rate (s^−1^) of A_1_B_1_ as a function of temperature *T* (K); and CS is the condensation sink (s^−1^) of A_1_B_1_ contributed by aerosols and H_2_SO_4_-amine clusters. The growth of A_1_B_1_, e.g. the clustering between two A_1_B_1_ molecules, is herein accounted for in CS. In urban Beijing and Shanghai, background aerosols contribute majorly to this CS term [37,61].

The overall lifetime of A_1_B_1_, *τ* (s), can be estimated using Equation (1). *τ* characterizes the typical time A_1_B_1_ concentration takes to reach its pseudo-steady-state value and it is equal to 1/(*γ*(*T*) + CS). For instance, by setting the source term in Equation (1) to zero at the moment *t*_0_ and keeping *γ*(*T*) + CS constant, [A_1_B_1_] at *t*_0_ + *τ* will reduce to 1/e of its concentration at *t*_0_. Considering the high CS for polluted environments, *τ* is usually <10 min. Hence, A_1_B_1_ is close to its pseudo-steady-state concentration regardless of a fast variation of atmospheric H_2_SO_4_ production rate or H_2_SO_4_ monomer concentration (Fig. S6).

Setting d[A_1_B_1_]/d*t* in Equation (1) to zero yields the formula for the pseudo-steady-state value of *η* [62]:

(2) \begin{eqnarray*} \eta &=& \frac{{\left[ {{{\rm{A}}}_1{{\rm{B}}}_1} \right]}}{{\left[ {{{\rm{A}}}_{1,{\rm{tot}}}} \right]}}\nonumber\\ &=& \frac{{\left[ {{{\rm{A}}}_1{{\rm{B}}}_1} \right]}}{{\left[ {{{\rm{A}}}_1} \right] + \ \left[ {{{\rm{A}}}_1{{\rm{B}}}_1} \right]}} = \frac{{{\beta }_{{\rm{AB}}}\left[ {{{\rm{B}}}_1} \right]}}{{{\beta }_{{\rm{AB}}}\left[ {{{\rm{B}}}_1} \right] + \gamma \left( T \right) + {\rm{CS}}}}.\nonumber\\ \end{eqnarray*}

The amine-saturation limit is the formation rate of clusters or particles with no evaporation and a unity coagulation efficiency. In this study, we refer to the amine-saturation limit as that for H_2_SO_4_ monomers corresponding to a sufficient base concentration. Accordingly, the H_2_SO_4_ dimer concentration at the amine-saturation limit can be calculated using Equation (3),

(3) \begin{equation*} {\left [{{{\rm{A}}}_{2,{\rm{tot}}}} \right]}_{{\rm{AS}}}\ = \ \frac{{{\beta }_{11}{{\left[ {{{\rm{A}}}_{1,{\rm{tot}}}} \right]}}^2}}{{2{\rm{CS}}}},\ \end{equation*}

where [A_1,tot_] and [A_2,tot_] are H_2_SO_4_ monomer and dimer concentrations (cm^−3^), respectively; the subscript AS stands for amine-saturation limit; [A_1,tot_] = [A_1_] + [A_1_B_1_]; *β*_11_ is the coagulation coefficient (cm^3^ s^−1^) between two H_2_SO_4_ monomers; and CS is the condensation sink (s^−1^) for H_2_SO_4_ monomers.

The amine-saturation limit of [A_2,tot_] is reached at an infinite [B_1_], with which *η* is equal to 1.0 according to Equation (2). Similarly, the amine-saturation limit of the particle formation rate is also reached at an infinite [B_1_]. In this study, the amine-saturation limits of [A_2,tot_] and the particle formation rate were obtained by setting the DMA concentration at 10^6^ ppt so that nucleation was not limited by DMA concentration.

The [A_2,tot_] and particle formation rates in Figs 3 and 4 were compared to their corresponding amine-saturation limits. With DMA as the base, and considering the high evaporation rate of neutral A_2_ and the negligible evaporation rates of A_2_D_1-2_ [22,36], it can be approximated that only monomer collisions involving at least one A_1_D_1_ (i.e. A_1_D_1 _+ A_1_ or A_1_D_1 _+ A_1_D_1_) form a stable dimer. Hence, the [A_2,tot_] for H_2_SO_4_-DMA nucleation can be approximated with *η* using Equation (4):

(4) \begin{eqnarray*} \left[ {{{\rm{A}}}_{2,{\rm{tot}}}} \right] &=& \left[ {{\eta }^2 + 2\eta \left( {1 - \eta } \right)} \right]{\left[ {{{\rm{A}}}_{2,{\rm{tot}}}} \right]}_{{\rm{AS}}}\nonumber\\ &=& \eta \left( {2 - \eta } \right){\left[ {{{\rm{A}}}_{2,{\rm{tot}}}} \right]}_{{\rm{AS}}}. \end{eqnarray*}

Equation (4) shows that [A_2,tot_] increases monotonically with *η* within the domain of *η*, and [A_2,tot_] = [A_2,tot_]_AS_ at *η* = 1. Hence, the measured [A_2,tot_]/[A_2,tot_]_AS_ can be used to indicate *η*. Note that to obtain better accuracy, we calculated the results in Figs 3 and 4 numerically instead of using Equation (4). Considering these minor differences, [A_2,tot_] may slightly exceed [A_2,tot_]_AS_ because the amine-saturation limit in Equation (3) is calculated using A_1_D_1_ and the thermal velocity of A_1_ is slightly higher than that of A_1_D_1_.

A process model is used to simulate the growth of H_2_SO_4_ clusters. The simulated bases, B, include ammonia, MA, DMA and TMA. The outputs of this model are the cluster concentrations and formation rate. This model has been reported previously [37] and similar models can be found in the literature [18,32]. Only the neutral nucleation mechanism is accounted for in this model because the ion production rate is not comparable to the high NPF rate in urban Beijing and Shanghai. The formation rate of H_2_SO_4_ tetramers was taken as the simulated NPF rate [18] because the electrical mobility diameter of H_2_SO_4_ tetramers was estimated to be 1.4 nm according to previous studies [63,64]. There are potential uncertainties caused by the difference between diameters for the measured and simulated particle formation rates, yet these uncertainties do not influence the conclusions in this analysis based on the temperature dependence of the particle formation rate.

The temperature-dependent evaporation rates of A_1_D_1_ were estimated by fitting Equations (2) and (4) to the data set of urban Beijing. We fitted the evaporation rate (corresponding to standard Gibbs free energy) at 298 K to minimize the residue of [A_2,tot_]/[A_2,tot_]_AS_ as shown in Fig. 2, whereas the temperature dependence of standard Gibbs free energy (corresponding to evaporation rate) was calculated using enthalpy given by quantum chemical calculations [22]. That is, we fitted the absolute value of the evaporation rate but not its temperature dependence. The evaporation rates of other clusters used in this process model were calculated using the standard Gibbs free energy given by quantum chemical calculations [21,22]. As shown in Fig. S4, the experimentally determined evaporation rates of A_1_D_1_ are within the uncertainty range of quantum chemical results and this uncertainty does not affect the findings. A coagulation enhancement factor due to Van der Waals force [65–67] was accounted for in the calculation of coagulation coefficients and evaporation rates. The species included in the process model were determined according to the evaporation rates. For instance, A_n_D_n+1_ clusters were reported to be unstable against evaporation in various quantum chemical results [22,36] and hence they are not included in the model.

The free energy barrier shown in Fig. 5 was calculated using standard Gibbs free energy and the measured concentrations of acid and base vapors [7]. For an A_m_B_n_ cluster, the formula for its free energy is given in Equation (5),

(5) \begin{eqnarray*} \Delta G\left( {{{\rm{A}}}_{\rm{m}}{{\rm{B}}}_{\rm{n}},T} \right) &=& \Delta {G}^{\rm{\theta }}\left( {{{\rm{A}}}_{\rm{m}}{{\rm{B}}}_{\rm{n}},T} \right)\\ && -\, \left( {{\rm{m}} - 1} \right)RT{\rm{ln}}\frac{{{P}_{\rm{A}}}}{{{P}_{{\rm{ref}}}}}\\ && -\, nRT{\rm{ln}}\frac{{{P}_{\rm{B}}}}{{{P}_{{\rm{ref}}}}}, \end{eqnarray*}

where $\Delta G$ is the free energy barrier (also named formation free energy in some nucleation studies); $\Delta {G}^{\rm{\theta }}$ (kcal mol^−1^) is the standard formation free energy (also named binding energy in some nucleation studies); *T* (K) is temperature; *R* is the ideal gas constant; *P*_A_ and *P*_B_ are the partial pressures of A_1_ and B_1_, respectively; and *P*_ref_ is the reference pressure used for calculating ${\Delta }_{\rm{f}}{G}^{\rm{\theta }}$. ${\Delta }_{\rm{f}}G$ characterizes the energy barrier for a bare sulfuric acid molecule A_1_ to form a certain cluster. The free energy of A_1_ is accordingly equal to zero. A positive free energy of A_1_B_1_ indicates that the association between A_1_ and B_1_ needs to overcome an energy barrier.

## DATA AVAILABILITY

Data can be found at https://doi.org/10.5281/zenodo.6801940. The Julia programming codes for the kinetic model in this study are available from the corresponding author upon request.

## Supplementary Material

nwac137_Supplemental_file

## References

[1] Kerminen V-M , Chen X, Vakkari V et al. Atmospheric new particle formation and growth: review of field observations. Environ Res Lett 2018; 13: 103003. 10.1088/1748-9326/aadf3c

[2] Nieminen T , Kerminen V-M, Petäjä T et al. Global analysis of continental boundary layer new particle formation based on long-term measurements. Atmos Chem Phys 2018; 18: 14737–56. 10.5194/acp-18-14737-2018

[3] Kuang C , McMurry PH, McCormick AV. Determination of cloud condensation nuclei production from measured new particle formation events. Geophys Res Lett 2009; 36: L09822. 10.1029/2009GL037584

[4] Gordon H , Kirkby J, Baltensperger U et al. Causes and importance of new particle formation in the present-day and preindustrial atmospheres. J Geophys Res Atmos 2017; 122: 8739–60. 10.1002/2017JD026844

[5] IPCC . Climate Change 2013: IPCC Fifth Assessment Report (AR5). Cambridge: Cambridge University Press, 2013.

[6] Zhang R , Khalizov A, Wang L et al. Nucleation and growth of nanoparticles in the atmosphere. Chem Rev 2012; 112: 1957–2011. 10.1021/cr2001756 22044487

[7] Elm J , Kubečka J, Besel V et al. Modeling the formation and growth of atmospheric molecular clusters: a review. J Aerosol Sci 2020; 149: 105621. 10.1016/j.jaerosci.2020.105621

[8] Almeida J , Schobesberger S, Kurten A et al. Molecular understanding of sulphuric acid-amine particle nucleation in the atmosphere. Nature 2013; 502: 359–63. 10.1038/nature12663 24097350 PMC7449521

[9] Chen M , Titcombe M, Jiang J et al. Acid-base chemical reaction model for nucleation rates in the polluted atmospheric boundary layer. Proc Natl Acad Sci USA 2012; 109: 18713–8. 10.1073/pnas.1210285109 23091030 PMC3503223

[10] Yao L , Garmash O, Bianchi F et al. Atmospheric new particle formation from sulfuric acid and amines in a Chinese megacity. Science 2018; 361: 278–81. 10.1126/science.aao4839 30026225

[11] Kirkby J , Curtius J, Almeida J et al. Role of sulphuric acid, ammonia and galactic cosmic rays in atmospheric aerosol nucleation. Nature 2011; 476: 429–33. 10.1038/nature10343 21866156

[12] Jokinen T , Sipilä M, Kontkanen J et al. Ion-induced sulfuric acid–ammonia nucleation drives particle formation in coastal Antarctica. Sci Adv 2018; 4: eaat9744. 10.1126/sciadv.aat9744 30498779 PMC6261657

[13] Kirkby J , Duplissy J, Sengupta K et al. Ion-induced nucleation of pure biogenic particles. Nature 2016; 533: 521–6. 10.1038/nature17953 27225125 PMC8384037

[14] Bianchi F , Tröstl J, Junninen H et al. New particle formation in the free troposphere: a question of chemistry and timing. Science 2016; 352: 1109–12. 10.1126/science.aad5456 27226488

[15] Riccobono F , Schobesberger S, Scott CE et al. Oxidation products of biogenic emissions contribute to nucleation of atmospheric particles. Science 2014; 344: 717–21. 10.1126/science.1243527 24833386

[16] Sipilä M , Sarnela N, Jokinen T et al. Molecular-scale evidence of aerosol particle formation via sequential addition of HIO_3_. Nature 2016; 537: 532–4. 10.1038/nature19314 27580030 PMC5136290

[17] He X-C , Tham YJ, Dada L et al. Role of iodine oxoacids in atmospheric aerosol nucleation. Science 2021; 371:589–95. 10.1126/science.abe0298 33542130

[18] Jen CN , McMurry PH, Hanson DR. Stabilization of sulfuric acid dimers by ammonia, methylamine, dimethylamine, and trimethylamine. J Geophys Res Atmos 2014; 119: 7502–14. 10.1002/2014JD021592

[19] Jen CN , Bachman R, Zhao J et al. Diamine-sulfuric acid reactions are a potent source of new particle formation. Geophys Res Lett 2016; 43: 867–73. 10.1002/2015GL066958

[20] Kürten A , Jokinen T, Simon M et al. Neutral molecular cluster formation of sulfuric acid-dimethylamine observed in real time under atmospheric conditions. Proc Natl Acad Sci USA 2014; 111: 15019–24. 10.1073/pnas.1404853111 25288761 PMC4210346

[21] Olenius T , Halonen R, Kurtén T et al. New particle formation from sulfuric acid and amines: comparison of monomethylamine, dimethylamine, and trimethylamine. J Geophys Res Atmos 2017; 122: 7103–18. 10.1002/2017JD026501

[22] Myllys N , Kubečka J, Besel V et al. Role of base strength, cluster structure and charge in sulfuric-acid-driven particle formation. Atmos Chem Phys 2019; 19: 9753–68. 10.5194/acp-19-9753-2019

[23] Hemmilä M , Hellén H, Virkkula A et al. Amines in boreal forest air at SMEAR II station in Finland. Atmos Chem Phys 2018; 18: 6367–80. 10.5194/acp-18-6367-2018

[24] Bianchi F , Praplan AP, Sarnela N et al. Insight into acid-base nucleation experiments by comparison of the chemical composition of positive, negative, and neutral clusters. Environ Sci Technol 2014; 48: 13675–84. 10.1021/es502380b 25406110

[25] Petäjä T , Sipilä M, Paasonen P et al. Experimental observation of strongly bound dimers of sulfuric acid: application to nucleation in the atmosphere. Phys Rev Lett 2011; 106: 228302. 10.1103/PhysRevLett.106.228302 21702637

[26] Chee S , Barsanti K, Smith JN et al. A predictive model for salt nanoparticle formation using heterodimer stability calculations. Atmos Chem Phys 2021; 21: 11637–54. 10.5194/acp-21-11637-2021

[27] Kurtén T , Petäjä T, Smith J et al. The effect of H_2_SO_4_ – amine clustering on chemical ionization mass spectrometry (CIMS) measurements of gas-phase sulfuric acid. Atmos Chem Phys 2011; 11: 3007–19. 10.5194/acp-11-3007-2011

[28] Kürten A , Li C, Bianchi F et al. New particle formation in the sulfuric acid–dimethylamine–water system: reevaluation of CLOUD chamber measurements and comparison to an aerosol nucleation and growth model. Atmos Chem Phys 2018; 18: 845–63. 10.5194/acp-18-845-2018

[29] Yin R , Yan C, Cai R et al. Acid-base clusters during atmospheric new particle formation in urban Beijing. Environ Sci Technol 2021; 55: 10994–1005. 10.1021/acs.est.1c02701 34338506

[30] Zapadinsky E , Passananti M, Myllys N et al. Modeling on fragmentation of clusters inside a mass spectrometer. J Phys Chem A 2019; 123: 611–24. 10.1021/acs.jpca.8b10744 30550283 PMC6340130

[31] Lehtipalo K , Yan C, Dada L et al. Multicomponent new particle formation from sulfuric acid, ammonia, and biogenic vapors. Sci Adv 2018; 4: eaau5363. 10.1126/sciadv.aau5363 30547087 PMC6291317

[32] McGrath MJ , Olenius T, Ortega IK et al. Atmospheric cluster dynamics code: a flexible method for solution of the birth-death equations. Atmos Chem Phys 2012; 12: 2345–55. 10.5194/acp-12-2345-2012

[33] Li C , Signorell R. Understanding vapor nucleation on the molecular level: a review. J Aerosol Sci 2021; 153: 105676. 10.1016/j.jaerosci.2020.105676

[34] Kürten A , Münch S, Rondo L et al. Thermodynamics of the formation of sulfuric acid dimers in the binary (H_2_SO_4_–H_2_O) and ternary (H_2_SO_4_–H_2_O–NH_3_) system. Atmos Chem Phys 2015; 15: 10701–21. 10.5194/acp-15-10701-2015

[35] Jen CN , Zhao J, McMurry PH et al. Chemical ionization of clusters formed from sulfuric acid and dimethylamine or diamines. Atmos Chem Phys 2016; 16: 12513–29. 10.5194/acp-16-12513-2016

[36] Ortega IK , Kupiainen O, Kurtén T et al. From quantum chemical formation free energies to evaporation rates. Atmos Chem Phys 2012; 12: 225–35. 10.5194/acp-12-225-2012

[37] Cai R , Yan C, Yang D et al. Sulfuric acid-amine nucleation in urban Beijing. Atmos Chem Phys 2021; 21: 2457–68. 10.5194/acp-21-2457-2021

[38] Yan C , Yin R, Lu Y et al. The synergistic role of sulfuric acid, bases, and oxidized organics governing new-particle formation in Beijing. Geophys Res Lett 2021; 48: e2020GL091944. 10.1029/2020GL091944

[39] Paasonen P , Olenius T, Kupiainen O et al. On the formation of sulphuric acid-amine clusters in varying atmospheric conditions and its influence on atmospheric new particle formation. Atmos Chem Phys 2012; 12: 9113–33. 10.5194/acp-12-9113-2012

[40] Ortega IK , Olenius T, Kupiainen-Määttä O et al. Electrical charging changes the composition of sulfuric acid–ammonia/dimethylamine clusters. Atmos Chem Phys 2014; 14: 7995–8007. 10.5194/acp-14-7995-2014

[41] Myllys N , Chee S, Olenius T et al. Molecular-level understanding of synergistic effects in sulfuric acid-amine-ammonia mixed clusters. J Phys Chem A 2019; 123: 2420–5. 10.1021/acs.jpca.9b00909 30821984

[42] Glasoe WA , Volz K, Panta B et al. Sulfuric acid nucleation: an experimental study of the effect of seven bases. J Geophys Res Atmos 2015; 120: 1933–50. 10.1002/2014JD022730

[43] Olenius T , Kupiainen-Maatta O, Ortega IK et al. Free energy barrier in the growth of sulfuric acid-ammonia and sulfuric acid-dimethylamine clusters. J Chem Phys 2013; 139: 084312. 10.1063/1.4819024 24007002

[44] Kupiainen O , Ortega IK, Kurtén T et al. Amine substitution into sulfuric acid-ammonia clusters. Atmos Chem Phys 2012; 12: 3591–9. 10.5194/acp-12-3591-2012

[45] Merikanto J , Duplissy J, Määttänen A et al. Effect of ions on sulfuric acid-water binary particle formation: 1. Theory for kinetic- and nucleation-type particle formation and atmospheric implications. J Geophys Res Atmos 2016; 121: 1736–51. 10.1002/2015JD023538

[46] Lehtipalo K , Rondo L, Kontkanen J et al. The effect of acid-base clustering and ions on the growth of atmospheric nano-particles. Nat Commun 2016; 7: 11594. 10.1038/ncomms11594 27197574 PMC4876472

[47] Jokinen T , Sipilä M, Junninen H et al. Atmospheric sulphuric acid and neutral cluster measurements using CI-APi-TOF. Atmos Chem Phys 2012; 12: 4117–25. 10.5194/acp-12-4117-2012

[48] Zheng J , Ma Y, Chen M et al. Measurement of atmospheric amines and ammonia using the high resolution time-of-flight chemical ionization mass spectrometry. Atmos Environ 2015; 102: 249–59. 10.1016/j.atmosenv.2014.12.002

[49] Kurtén T , Loukonen V, Vehkamäki H et al. Amines are likely to enhance neutral and ion-induced sulfuric acid-water nucleation in the atmosphere more effectively than ammonia. Atmos Chem Phys 2008; 8: 4095–103. 10.5194/acp-8-4095-2008

[50] Jiang J , Chen M, Kuang C et al. Electrical mobility spectrometer using a diethylene glycol condensation particle counter for measurement of aerosol size distributions down to 1 nm. Aerosol Sci Technol 2011; 45: 510–21. 10.1080/02786826.2010.547538

[51] Cai R , Chen D-R, Hao J et al. A miniature cylindrical differential mobility analyzer for sub-3 nm particle sizing. J Aerosol Sci 2017; 106: 111–9. 10.1016/j.jaerosci.2017.01.004

[52] Fu Y , Xue M, Cai R et al. Theoretical and experimental analysis of the core sampling method: reducing diffusional losses in aerosol sampling line. Aerosol Sci Technol 2019; 53: 793–801. 10.1080/02786826.2019.1608354

[53] Liu J , Jiang J, Zhang Q et al. A spectrometer for measuring particle size distributions in the range of 3 nm to 10 μm. Front Environ Sci Eng 2016; 10: 63–72. 10.1007/s11783-014-0754-x

[54] Deng C , Fu Y, Dada L et al. Seasonal characteristics of new particle formation and growth in urban Beijing. Environ Sci Technol 2020; 54: 8547–57. 10.1021/acs.est.0c00808 32609510

[55] Liu Y , Yan C, Feng Z et al. Continuous and comprehensive atmospheric observations in Beijing: a station to understand the complex urban atmospheric environment. Big Earth Data 2020; 4: 295–321. 10.1080/20964471.2020.1798707

[56] Lehtipalo K , Leppä J, Kontkanen J et al. Methods for determining particle size distribution and growth rates between 1 and 3 nm using the Particle Size Magnifier. Boreal Environ Res 2014; 19: 215–36.

[57] Yao L , Wang M-Y, Wang X-K et al. Detection of atmospheric gaseous amines and amides by a high-resolution time-of-flight chemical ionization mass spectrometer with protonated ethanol reagent ions. Atmos Chem Phys 2016; 16: 14527–43. 10.5194/acp-16-14527-2016

[58] Heinritzi M , Simon M, Steiner G et al. Characterization of the mass-dependent transmission efficiency of a CIMS. Atmos Meas Tech 2016; 9: 1449–60. 10.5194/amt-9-1449-2016

[59] Cai R , Jiang J. A new balance formula to estimate new particle formation rate: reevaluating the effect of coagulation scavenging. Atmos Chem Phys 2017; 17: 12659–75. 10.5194/acp-17-12659-2017

[60] Kerminen VM , Kulmala M. Analytical formulae connecting the “real” and the “apparent” nucleation rate and the nuclei number concentration for atmospheric nucleation events. J Aerosol Sci 2002; 33: 609–22. 10.1016/S0021-8502(01)00194-X

[61] Cai R , Yang D, Fu Y et al. Aerosol surface area concentration: a governing factor in new particle formation in Beijing. Atmos Chem Phys 2017; 17: 12327–40. 10.5194/acp-17-12327-2017

[62] Cai R , Yan C, Worsnop DR et al. An indicator for sulfuric acid-amine nucleation in atmospheric environments. Aerosol Sci Technol 2021; 55: 1059–69. 10.1080/02786826.2021.1922598

[63] Jen CN , Hanson DR, McMurry PH. Toward reconciling measurements of atmospherically relevant clusters by chemical ionization mass spectrometry and mobility classification/vapor condensation. Aerosol Sci Technol 2015; 49:i–iii. 10.1080/02786826.2014.1002602

[64] Thomas JM , He S, Larriba-Andaluz C et al. Ion mobility spectrometry-mass spectrometry examination of the structures, stabilities, and extents of hydration of dimethylamine-sulfuric acid clusters. Phys Chem Chem Phys 2016; 18: 22962–72. 10.1039/C6CP03432B 27485283

[65] Chan TW , Mozurkewich M. Measurement of the coagulation rate constant for sulfuric acid particles as a function of particle size using tandem differential mobility analysis. J Aerosol Sci 2001; 32: 321–39. 10.1016/S0021-8502(00)00081-1

[66] Stolzenburg D , Simon M, Ranjithkumar A et al. Enhanced growth rate of atmospheric particles from sulfuric acid. Atmos Chem Phys 2020; 20: 7359–72. 10.5194/acp-20-7359-2020

[67] Halonen R , Zapadinsky E, Kurtén T et al. Rate enhancement in collisions of sulfuric acid molecules due to long-range intermolecular forces. Atmos Chem Phys 2019; 19: 13355–66. 10.5194/acp-19-13355-2019

[68] Deng C , Cai R, Yan C et al. Formation and growth of sub-3 nm particles in megacities: impacts of background aerosols. Farad Discuss 2021; 226: 348–63. 10.1039/D0FD00083C 33237099

