# Supplementary material for: The missing base molecules in atmospheric acid–base nucleation
Source: Natl Sci Rev. 2022 Jul 25;9(10):nwac137. doi: 10.1093/nsr/nwac137 (PMC9522409; doi:10.1093/nsr/nwac137)
Supplement: nwac137_Supplemental_file [file nwac137_supplemental_file.pdf]

Supplementary Information for

The missing base molecules in atmospheric acid-base nucleation

Runlong Cai[a,b], Rujing Yin[a], Chao Yan[b,c], Dongsen Yang[d], Chenjuan Deng[a], Lubna Dada[b,e], Juha Kangasluoma[b], Jenni Kontkanen[b], Roope Halonen[f], Yan Ma[d], Xiuhui Zhang[g], Pauli Paasonen[b], Tuukka Petäjä[b], Veli-Matti Kerminen[b], Yongchun Liu[c], Federico Bianchi[b], Jun Zheng[d], Lin Wang[h], Jiming Hao[a], James N. Smith[i], Neil M. Donahue[j,k], Markku Kulmala[b,*], Douglas R. Worsnop[b,l], Jingkun Jiang[a,*]

[a]State Key Joint Laboratory of Environment Simulation and Pollution Control, School of Environment, Tsinghua University, Beijing, 100084, China

[b]Institute for Atmospheric and Earth System Research / Physics, Faculty of Science, University of Helsinki, Helsinki, 00014, Finland

[c]Aerosol and Haze Laboratory, Beijing Advanced Innovation Center for Soft Matter Science and Engineering, Beijing University of Chemical Technology, Beijing, 100029, China

[d]Collaborative Innovation Center of Atmospheric Environment and Equipment Technology, Nanjing University of Information Science and Technology, Nanjing, 210044, China

[e]Laboratory of Atmospheric Chemistry, Paul Scherrer Institute, Villigen, 5232, Switzerland

[f]Center for Joint Quantum Studies and Department of Physics, School of Science, Tianjin University, 135 Yaguan Road, Tianjin, 300350, China

[g]Key Laboratory of Cluster Science, Ministry of Education of China, School of Chemistry and Chemical Engineering, Beijing Institute of Technology, Beijing, 100081, China

[h]Shanghai Key Laboratory of Atmospheric Particle Pollution and Prevention (LAP^3), Department of Environmental Science and Engineering, Fudan University, Shanghai, 200433, China

[i]Chemistry Department, University of California, Irvine, CA 92697, USA

[j]Center for Atmospheric Particle Studies, Carnegie Mellon University, Pittsburgh, PA 15213, USA

[k]Department of Chemistry, Carnegie Mellon University, Pittsburgh, PA 15213, USA

[l]Aerodyne Research Inc., Billerica, Massachusetts, MA 01821, USA

*Jingkun Jiang and Markku Kulmala

Email: jiangjk@tsinghua.edu.cn; markku.kulmala@helsinki.fi

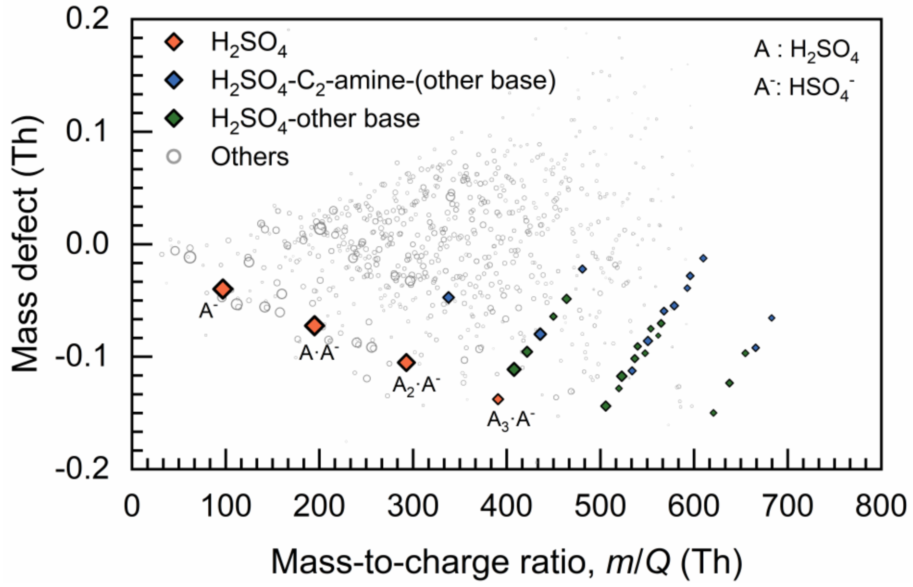

Figure S1: Naturally charged molecules and clusters measured during ambient new particle formation events from H_2SO_4 and amines

The negatively charged ambient molecules and clusters measured using atmospheric pressure interface mass spectrometry in urban Beijing. No bases were detected in the naturally charged H_2SO_4 monomers and dimers. The identified bases in larger H_2SO_4 clusters include C_1-C_4 amines and ammonia. Detailed discussions on the composition of naturally charged H_2SO_4 clusters can be found in Ref. [1].

The sizes of H_2SO_4 clusters and other species indicate their signal intensities, yet they follow a different size scale in order to emphasize the H_2SO_4 clusters.

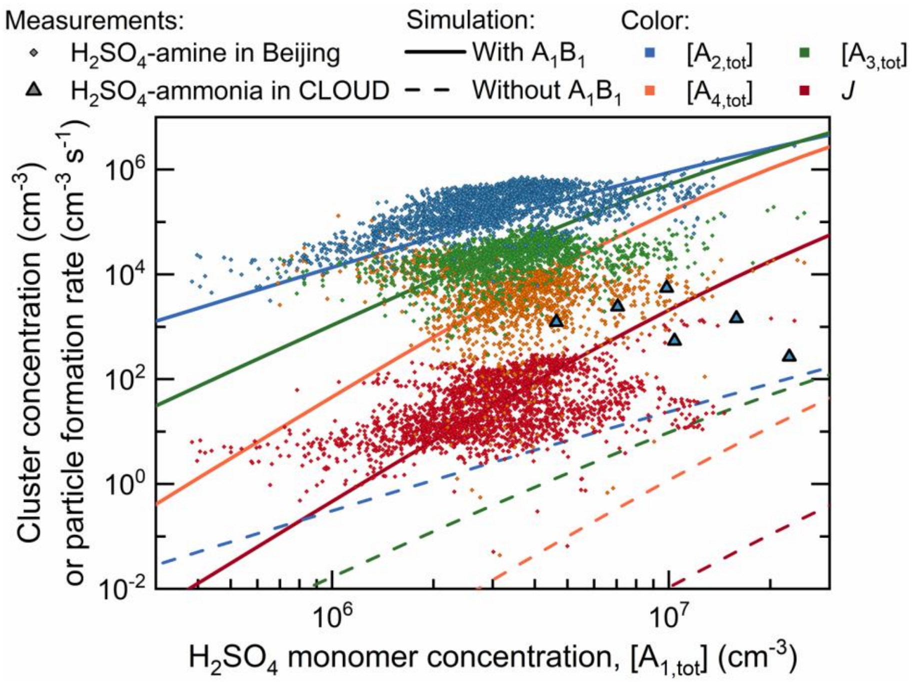

Figure S2: Scaled H_2SO_4 cluster concentrations and particle formation rates in urban Beijing and H_2SO_4 dimer concentration in an H_2SO_4-NH_3-H_2O system

The data measured in Beijing are scaled to the median condensation sink (CS) to minimize the influence of the correlation between CS and $[A_{1,tot}]$ on the slope of cluster concentration or J versus $[A_{1,tot}]$. The scaling formula is $X_{scaled} = X_{raw} \times (CS/CS_{median})^n$, where X is cluster concentration or J [2]. The value of n is 1, 2, 3, and 2 when X is $[A_{2,tot}]$, $[A_{3,tot}]$, $[A_{4,tot}]$, and J, respectively. The measured trimer and tetramer concentrations may be underestimated due to their uncorrected low detection efficiencies [3], hence they are lower than the simulated values. The simulation curves are identical to those in Fig. 2 in the main text. This figure shows that after correcting the influence of CS, the simulated trends of cluster concentrations and J as a function of $[A_{1,tot}]$ are consistent with the measured trends.

The data for H_2SO_4-ammonia-water nucleation from CLOUD chamber experiments was reported in [4]. As shown in Fig. 5 in the main text, $(H_2SO_4)_1(\text{ammonia})_1$ is far less stable than $(H_2SO_4)_1(\text{DMA})_1$, and therefore most H_2SO_4 monomers are supposed to exist in the form of bare H_2SO_4 molecules. However, $(H_2SO_4)_2(\text{ammonia})_1$ are stable against evaporation at the experimental conditions (278 K) according to quantum chemical results [5], indicating that A_2 can be rapidly stabilized ammonia. Despite the higher ammonia concentration and lower CS in the experiments compared to the DMA concentration and CS in the simulation, the measured $[A_{2,tot}]$ in the CLOUD chamber with H_2SO_4 and ammonia is substantially lower than the simulation curve via A_1D_1. This provides support for the importance of A_1D_1 to the formation of stable H_2SO_4 dimers.

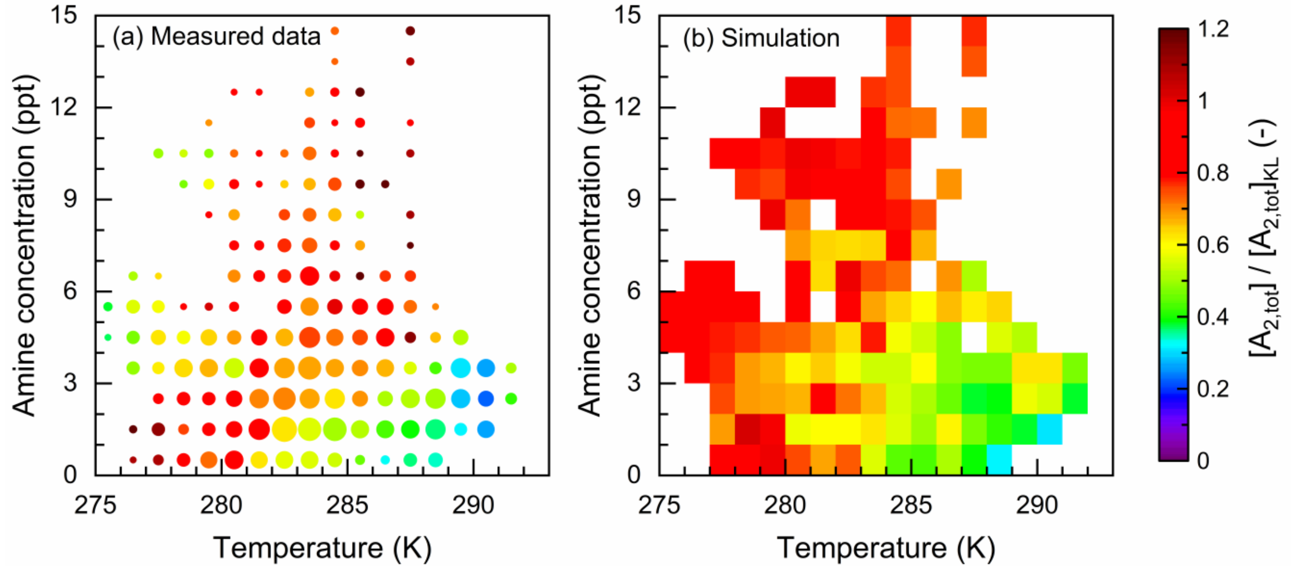

Figure S3: H_2SO_4 dimer concentration ($[A_{2,tot}]$) as a function of amine concentration and temperature for urban Beijing. The color indicates the deviation of $[A_{2,tot}]$ from its theoretical amine-saturation limit ($[A_{2,tot}]_{AS}$) due to the evaporation of A_1B_1 clusters. The amine-saturation limit assumes that each collision between two H_2SO_4 monomers forms a stable H_2SO_4 dimer.

a) Measured data. The markers show the median values of measured data grouped by temperature and amine concentration and their sizes indicate the number of measured data points in each grid. Note that there may be uncertainties in the measured data.

b) Simulation results. The input condensation sink for each grid is the median value of the measured data in (a) within the corresponding amine concentration and temperature range. The uncertainty in the evaporation rate of A_1B_1 is the main source of the uncertainties in the simulation results.

Amine concentration is defined as [C_2-amine]+0.2[C_3-amine]. The factor of 0.2 originates from experimental results [6] showing that the evaporation of A_1T_1 is ~5 times higher than A_1D_1, where D and T represent dimethylamine and trimethylamine, respectively. Note that adding this 0.2[C_3-amine] term or not does not affect the conclusions because of the low concentration of C_3-amines and the good correlations between [C_2-amine] and [C_3-amine]. For this reason, we use [DMA] in Fig. 3 in the main text to clarify the illustration.

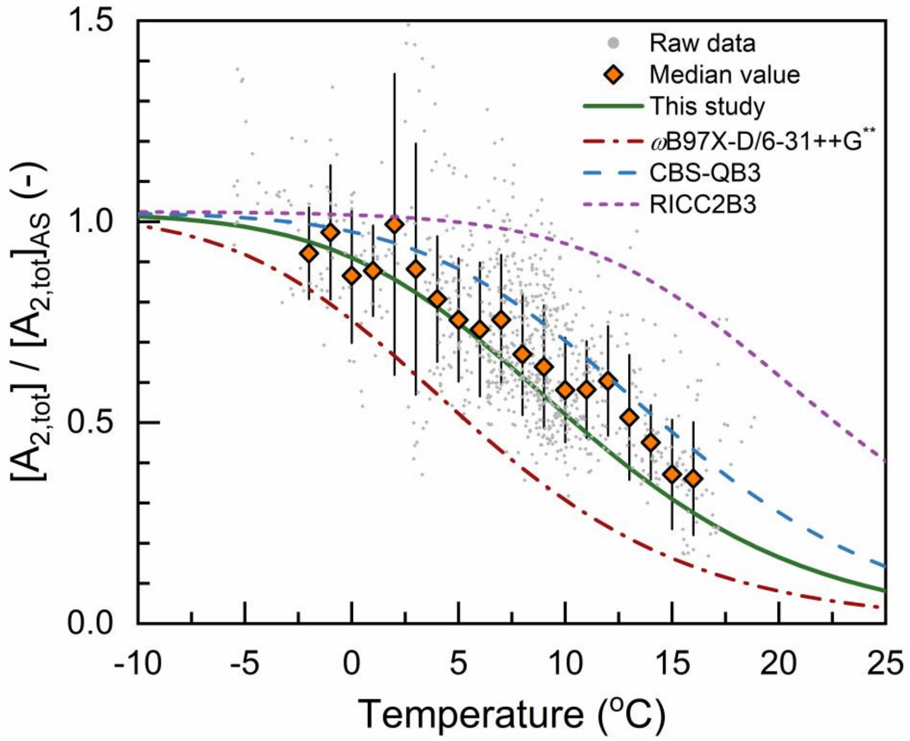

Figure S4: Temperature dependence of H_2SO_4 dimer concentration ([$A_{2,tot}$]) with different cluster evaporation rates. [$A_{2,tot}$] is normalized by dividing it by the H_2SO_4 dimer concentration at the amine-saturation limit ([$A_{2,tot}$]$_{AS}$). This figure shows that considering the uncertainty of quantum chemistry results for the Gibbs free energy, the measured temperature dependence of [$A_{2,tot}$] is consistent with the process model.

The measured data shown in markers and the simulation curve labeled by "this study" are identical to those in Figure 4b. The evaporation rate of A_1D_1 (D for dimethylamine) is calculated from its standard formation free energy, $\Delta_f G_m^\theta$. The value of $\Delta_f G_m^\theta$(298 K) used in this study is -14.0 kcal/mol. The reported quantum chemistry results[7, 8] for $\Delta_f G_{m,A1B1}^\theta$(298 K) using the ωB97X-D/6-31++G**, CBS-QB3, and RICC2B3 level of theory are -13.5, -14.4, and -15.4 kcal/mol, respectively. These values show that the fitted $\Delta_f G_m^\theta$, and its corresponding evaporate rate, are within the uncertainty range of quantum chemistry results.

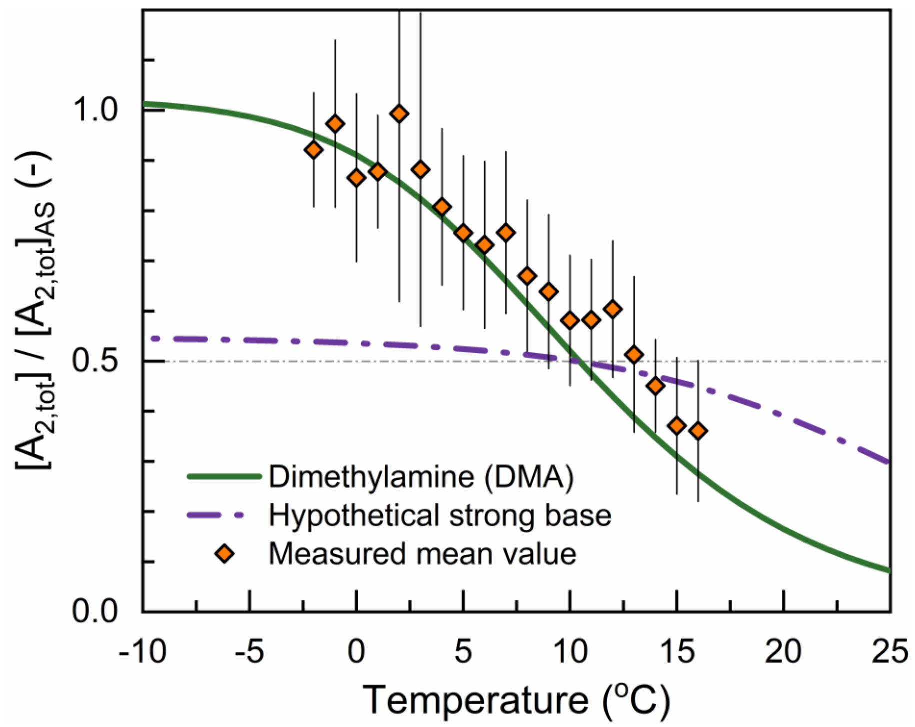

Figure S5: Temperature dependence of H_2SO_4 dimer concentration ($[A_{2,tot}]$) with DMA and an unidentified strong base. $[A_{2,tot}]$ is normalized by dividing it by the H_2SO_4 dimer concentration at the amine-saturation limit ($[A_{2,tot}]_{AS}$). The scatters and error bars indicate the measured data in urban Beijing, as illustrated in Figure 4. The simulation conditions and results for the DMA curve are identical to those in Figure 4b. This figure shows that the temperature dependency of H_2SO_4 dimer concentration with a stronger base is less significant than that with DMA. This is because the temperature dependence of dimer concentration, with DMA as the key base, is governed by the temperature-dependent evaporation rate of A_1D_1 (A for H_2SO_4 and D for DMA). In contrast, with a strong base, the coagulation loss rate is larger than the evaporation rate, hence the former limiting ambient dimer concentration without a significant temperature dependence. Therefore, the missing base in the measured H_2SO_4 monomers and dimers are more likely to be DMA than an unidentified strong base.

The normalized $[A_{2,tot}]$ is obtained by dividing $[A_{2,tot}]$ by its corresponding value at the amine-saturation limit, as elaborated in Figures 4 and S2. The evaporation rate of A_1B_1 (B for the unidentified strong base) is assumed to be 1% that of the evaporation rate of A_1D_1 at any given temperature. The concentration of this unidentified strong base is determined so that the H_2SO_4 dimer concentration reaches 50% of $[A_{2,tot}]_{AS}$ at the same temperature as the DMA curve.

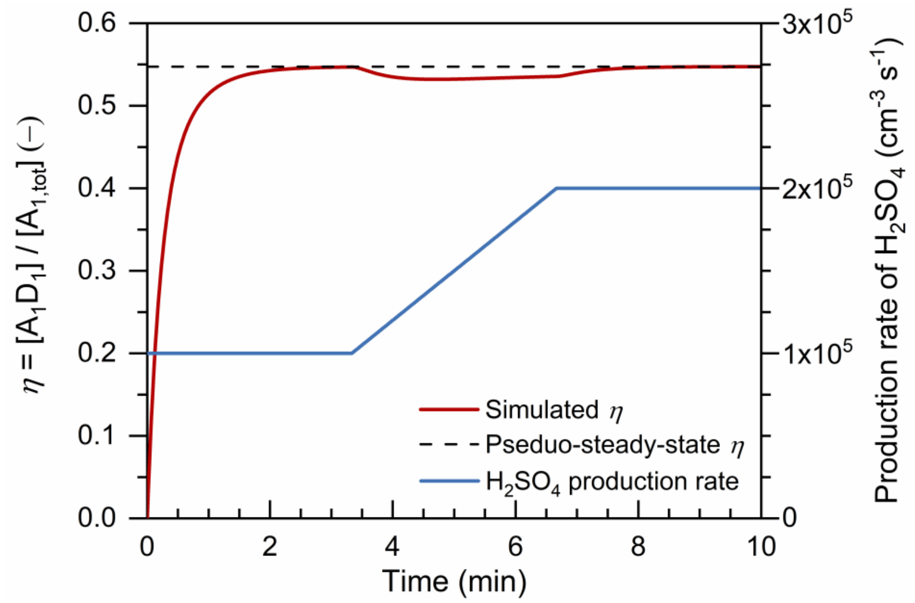

Figure S6: Proportion of A_1D_1 to total H_2SO_4 monomers as a function a time. A (acid) represents H_2SO_4, D represents DMA (dimethylamine), and η is the ratio of $[A_1D_1]$ to total H_2SO_4 monomer concentration ($[A_{1,tot}]$). The simulation results in this figure show that A_1D_1 reaches its pseudo-steady-state fraction within several minutes. Although the H_2SO_4 production rate doubled within 3 min (which is much faster than typical atmospheric processes), the A_1D_1 fraction in H_2SO_4 monomers reaches a steady-state value in less than 1 min and this value does not deviate much from the pseudo-steady-state value. Hence, it can be approximated that η is at a pseudo-steady-state during atmospheric new particle formation events.

The results for this figure were simulated at $[D_1]$ = 2.5 ppt, T = 273.15 K, and CS = 0.01 s^{-1} CS is the condensation sink of A_1D_1. The initial concentrations of A_1 and A_1D_1 were set to zero. The production rate of A_1 is given in the figure. The pseudo-steady-state value of η is given in Eq. 2 in the main text.

A potential concern is that with a high evaporate rate (γ) of A_1D_1, the A_1D_1 concentration may not reach its pseudo-steady-state value due to the low net clustering rate between A_1 and D_1. This net clustering rate is sometimes referred to as the arrival rate [3, 9] of D_1. However, with a fixed production rate of A_1, it can be demonstrated that A_1D_1 reaches its pseudo-steady-state concentration faster at higher γ and CS [10].

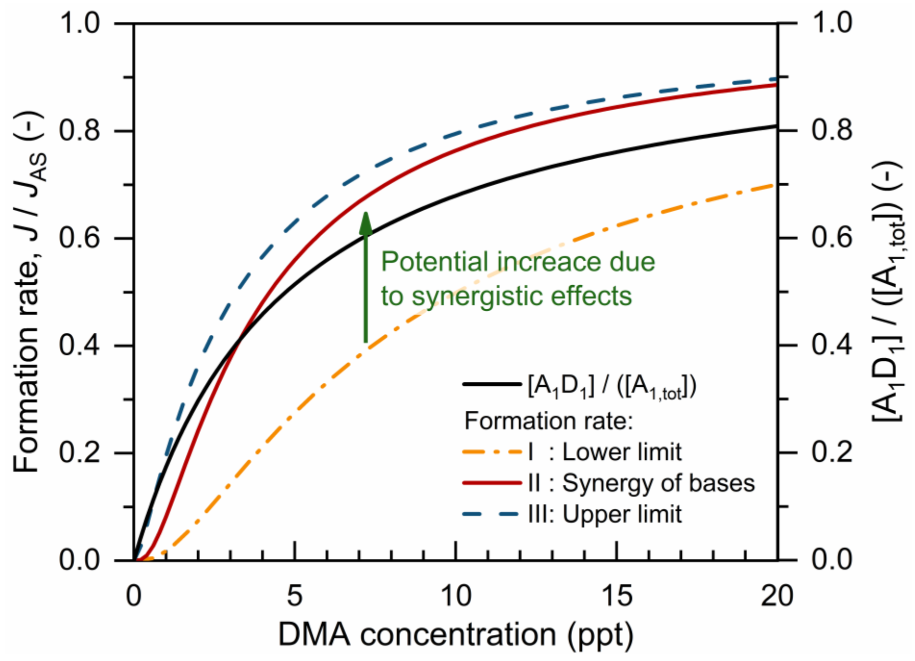

Figure S7: Possible contributions of the synergistic effects of dimethylamine (DMA) and ammonia to the new particle formation rate (*J*). The difference between curves I and II indicates that the synergy between NH_3 and DMA may significantly enhance particle formation rate, especially at a low DMA concentration.

A (acid) represents H_2SO_4 and D represents DMA. J_{AS} is the particle formation rate at the amine-saturation limit (see Figure 4). The simulation was performed for three parallel cases.

Case I: The evaporation rate of A_3D_2 and A_4D_3 are assumed to be high (10 s^{-1}) so that an A_nD_n cluster can hardly grow by the condensation of a bare A_1 molecule. Hence, only the clustering between A_nD_n and A_1D_1 contributes to cluster growth. This case represents a lower limit of the simulated particle formation rate.

Case II: the condensation rate of a D_1 molecule onto A_nD_{n-1} is enhanced by 500 times and it represents the synergy due to a high concentration of weak bases such as NH_3. The factor of 500 is taken from the approximate ratio of NH_3 concentration to C_2-amine concentrations measured in urban Beijing. In this case, a proportion of A_nD_{n-1} clusters are stabilized by NH_3 before they evaporate into $A_{n-1}D_{n-1}$.

Case III: A_nD_{n-1} clusters are assumed to be stable against evaporation and hence bare A_1 molecules contribute to cluster growth effectively. This case represents an upper limit of the simulated particle formation rate.

The simulation conditions are: $[A_{1,tot}] = 2.7\times10^6$ cm^{-3}, [D] = 1.8 ppt, condensation sink = 0.011 s^{-1}, and $T = 281$ K. The evaporation of A_2D_1 and A_nD_n ($n \geq 1$) are negligible according to quantum chemistry results [7].

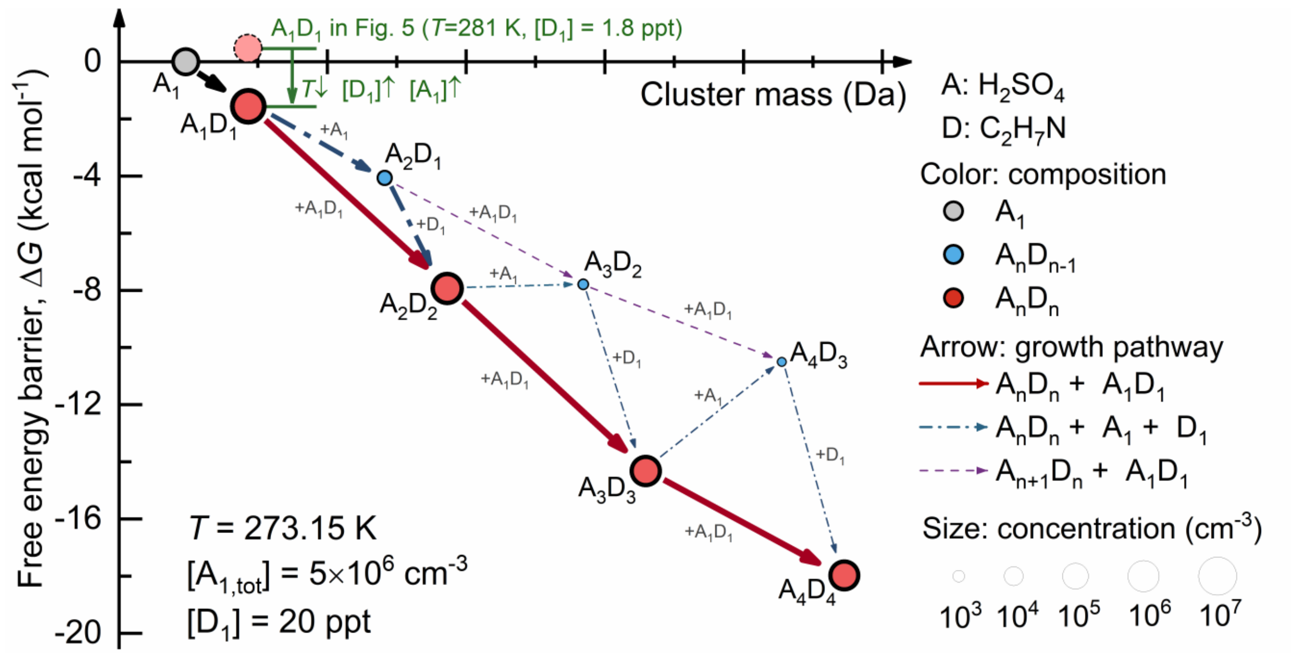

Figure S8: Compositions and Gibbs free energy of clusters during new particle formation from H_2SO_4 and amine at a high dimethylamine concentration and a low temperature. A (acid) represents H_2SO_4, D represents DMA (dimethylamine), and *T* is temperature. The conditions are: $[A_{1,tot}] = 5\times10^6$ cm^{-3}, $[D_1]$ = 20 ppt, condensation sink = 0.002 s^{-1}, and *T* = 273.15 K, which are close to the conditions for the CLOUD experiments reported in refs. (2) and (6). Compared to Figure 5, $[A_{1,tot}]$ and $[D_1]$ for this figure are higher and *T* for this figure is lower.

The free energy barrier [12] accounts for the concentrations of H_2SO_4 and DMA. Due to the high DMA concentration and the low temperature, the free energy of A_1D_1 for this figure is lower than that for Figure 5 and hence nucleation occurs without a free energy barrier. Under these conditions, most H_2SO_4 monomers exist in the form of A_1D_1 and hence the clustering between A_nD_n and A_1D_1 is the governing mechanism for the initial growth of H_2SO_4-DMA clusters [3, 11]. The growth pathway from A_1 to A_2D_1 is not shown because it is identical to the pathway from A_1D_1 to A_2D_1.

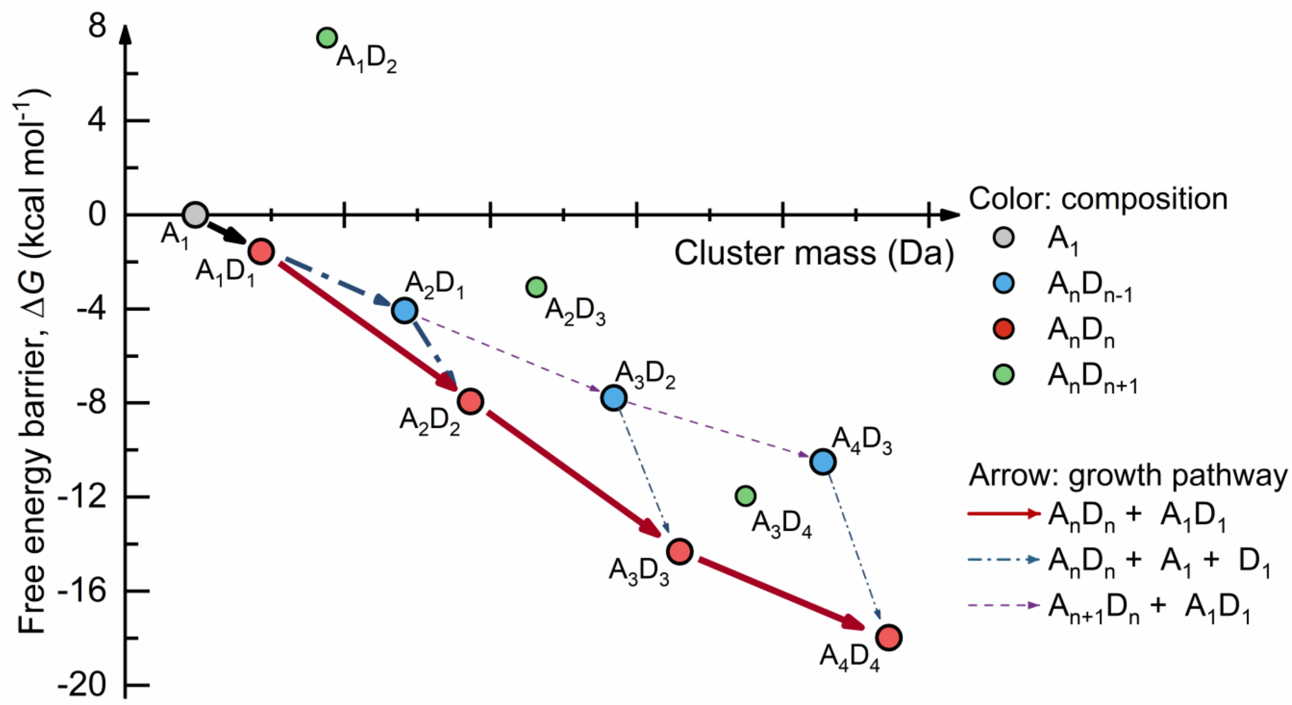

Figure S9: Gibbs free energy of A_nD_{n+1} clusters. The simulation conditions are identical to those in Fig. S8. Only the pathways with decreasing free energy are shown. This figure indicates that forming A_nD_{n+1} via $A_nD_n + A_1$ needs to overcome a positive free energy barrier, which is consequently not a major pathway for cluster growth.

References

1. Yin, R, Yan, C, Cai, R, *et al.* Acid-base clusters during atmospheric new particle formation in urban Beijing. *Environmental Science and Technology*. 2021; **55**(16): 10994–1005.
2. Cai, R, Yan, C, Yang, D, *et al.* Sulfuric acid-amine nucleation in urban Beijing. *Atmospheric Chemistry and Physics*. 2021; **21**: 2457–68.
3. Kürten, A, Jokinen, T, Simon, M, *et al.* Neutral molecular cluster formation of sulfuric acid-dimethylamine observed in real time under atmospheric conditions. *Proceedings of the National Academy of Sciences of the United States of America*. 2014; **111**(42): 15019-24.
4. Almeida, J, Schobesberger, S, Kurten, A, *et al.* Molecular understanding of sulphuric acid-amine particle nucleation in the atmosphere. *Nature*. 2013; **502**(7471): 359-63.
5. Myllys, N, Kubečka, J, Besel, V, *et al.* Role of base strength, cluster structure and charge in sulfuric-acid-driven particle formation. *Atmospheric Chemistry and Physics*. 2019; **19**(15): 9753-68.
6. Jen, CN, McMurry, PH, Hanson, DR. Stabilization of sulfuric acid dimers by ammonia, methylamine, dimethylamine, and trimethylamine. *Journal of Geophysical Research: Atmospheres*. 2014; **119**(12): 7502-14.
7. Myllys, N, Chee, S, Olenius, T, *et al.* Molecular-Level Understanding of Synergistic Effects in Sulfuric Acid-Amine-Ammonia Mixed Clusters. *J Phys Chem A*. 2019; **123**(12): 2420-5.
8. Ortega, IK, Kupiainen, O, Kurtén, T, *et al.* From quantum chemical formation free energies to evaporation rates. *Atmospheric Chemistry and Physics*. 2012; **12**(1): 225-35.
9. Bianchi, F, Praplan, AP, Sarnela, N, *et al.* Insight into acid-base nucleation experiments by comparison of the chemical composition of positive, negative, and neutral clusters. *Environ Sci Technol*. 2014; **48**(23): 13675-84.
10. Cai, R, Li, C, He, X-C, *et al.* Impacts of coagulation on the appearance time method for new particle growth rate evaluation and their corrections. *Atmospheric Chemistry and Physics*. 2021; **21**(3): 2287-304.
11. Kürten, A, Li, C, Bianchi, F, *et al.* New particle formation in the sulfuric acid–dimethylamine–water system: reevaluation of CLOUD chamber measurements and comparison to an aerosol nucleation and growth model. *Atmospheric Chemistry and Physics*. 2018; **18**(2): 845-63.
12. Elm, J, Kubečka, J, Besel, V, *et al.* Modeling the formation and growth of atmospheric molecular clusters: A review. *Journal of Aerosol Science*. 2020; **149**.
